# Effects of a Technology-Assisted Integrated Diabetes Care Program on Cardiometabolic Risk Factors Among Patients With Type 2 Diabetes in the Asia-Pacific Region: The JADE Program Randomized Clinical Trial

**DOI:** 10.1001/jamanetworkopen.2021.7557

**Published:** 2021-04-30

**Authors:** Lee-Ling Lim, Eric S. H. Lau, Amy W. C. Fu, Subir Ray, Yi-Jen Hung, Alexander T. B. Tan, Parinya Chamnan, Wayne H. H. Sheu, Manoj S. Chawla, Yook-Chin Chia, Lee-Ming Chuang, Duc-Cong Nguyen, Aravind Sosale, Banshi D. Saboo, Uday Phadke, Jothydev Kesavadev, Su-Yen Goh, Neeru Gera, Thi Thanh Huyen Vu, Ronald C. W. Ma, Vanessa Lau, Andrea O. Y. Luk, Alice P. S. Kong, Juliana C. N. Chan

**Affiliations:** 1 Department of Medicine and Therapeutics, The Chinese University of Hong Kong, Prince of Wales Hospital, Shatin, Hong Kong Special Administrative Region, China; 2 Asia Diabetes Foundation, Shatin, Hong Kong Special Administrative Region, China; 3 Department of Medicine, Faculty of Medicine, University of Malaya, Kuala Lumpur, Malaysia; 4 Relief Polyclinic, Hoogly, India; 5 Tri-Service General Hospital, Taipei, Taiwan; 6 Now with Sunway Medical Centre, Selangor, Malaysia; 7 Sanpasitthiprasong Hospital, Ubon Ratchathani, Thailand; 8 Taichung Veterans General Hospital, Taichung, Taiwan; 9 Lina Diabetes Care Centre, Mumbai, India; 10 Department of Primary Care Medicine, Faculty of Medicine, University of Malaya, Kuala Lumpur, Malaysia; 11 National Taiwan University Hospital, Taipei, Taiwan; 12 Thong Nhat Hospital, Ho Chi Minh, Vietnam; 13 Diacon Hospital, Bangalore, India; 14 Dia Care–Diabetes Care and Hormone Clinic, Gujarat, India; 15 Instride, Pune, India; 16 Jothydev’s Diabetes and Research Center, Kerala, India; 17 Department of Endocrinology, Singapore General Hospital, Outram Road, Singapore; 18 Max Healthcare Institute, New Delhi, India; 19 Department of Internal Medicine, Hanoi Medical University, Hanoi, Vietnam; 20 Hong Kong Institute of Diabetes and Obesity, The Chinese University of Hong Kong, Prince of Wales Hospital, Shatin, Hong Kong Special Administrative Region, China; 21 Li Ka Shing Institute of Health Sciences, The Chinese University of Hong Kong, Prince of Wales Hospital, Shatin, Hong Kong Special Administrative Region, China

## Abstract

**Importance:**

Many health care systems lack the efficiency, preparedness, or resources needed to address the increasing number of patients with type 2 diabetes, especially in low- and middle-income countries.

**Objective:**

To examine the effects of a quality improvement intervention comprising information and communications technology and contact with nonphysician personnel on the care and cardiometabolic risk factors of patients with type 2 diabetes in 8 Asia-Pacific countries.

**Design, Setting, and Participants:**

This 12-month multinational open-label randomized clinical trial was conducted from June 28, 2012, to April 28, 2016, at 50 primary care or hospital-based diabetes centers in 8 Asia-Pacific countries (India, Indonesia, Malaysia, the Philippines, Singapore, Taiwan, Thailand, and Vietnam). Six countries were low and middle income, and 2 countries were high income. The study was conducted in 2 phases; phase 1 enrolled 7537 participants, and phase 2 enrolled 13 297 participants. Participants in both phases were randomized on a 1:1 ratio to intervention or control groups. Data were analyzed by intention to treat and per protocol from July 3, 2019, to July 21, 2020.

**Interventions:**

In both phases, the intervention group received 3 care components: a nurse-led Joint Asia Diabetes Evaluation (JADE) technology-guided structured evaluation, automated personalized reports to encourage patient empowerment, and 2 or more telephone or face-to-face contacts by nurses to increase patient engagement. In phase 1, the control group received the JADE technology–guided structured evaluation and automated personalized reports. In phase 2, the control group received the JADE technology–guided structured evaluation only.

**Main Outcomes and Measures:**

The primary outcome was the incidence of a composite of diabetes-associated end points, including cardiovascular disease, chronic kidney disease, visual impairment or eye surgery, lower extremity amputation or foot ulcers requiring hospitalization, all-site cancers, and death. The secondary outcomes were the attainment of 2 or more primary diabetes-associated targets (glycated hemoglobin A_1c_ <7.0%, blood pressure <130/80 mm Hg, and low-density lipoprotein cholesterol <100 mg/dL) and/or 2 or more key performance indices (reduction in glycated hemoglobin A_1c_≥0.5%, reduction in systolic blood pressure ≥5 mm Hg, reduction in low-density lipoprotein cholesterol ≥19 mg/dL, and reduction in body weight ≥3.0%).

**Results:**

A total of 20 834 patients with type 2 diabetes were randomized in phases 1 and 2. In phase 1, 7537 participants (mean [SD] age, 60.0 [11.3] years; 3914 men [51.9%]; 4855 patients [64.4%] from low- and middle-income countries) were randomized, with 3732 patients allocated to the intervention group and 3805 patients allocated to the control group. In phase 2, 13 297 participants (mean [SD] age, 54.0 [11.1] years; 7754 men [58.3%]; 13 297 patients [100%] from low- and middle-income countries) were randomized, with 6645 patients allocated to the intervention group and 6652 patients allocated to the control group. In phase 1, compared with the control group, the intervention group had a similar risk of experiencing any of the primary outcomes (odds ratio [OR], 0.94; 95% CI, 0.74-1.21) but had an increased likelihood of attaining 2 or more primary targets (OR, 1.34; 95% CI, 1.21-1.49) and 2 or more key performance indices (OR, 1.18; 95% CI, 1.04-1.34). In phase 2, the intervention group also had a similar risk of experiencing any of the primary outcomes (OR, 1.02; 95% CI, 0.83-1.25) and had a greater likelihood of attaining 2 or more primary targets (OR, 1.25; 95% CI, 1.14-1.37) and 2 or more key performance indices (OR, 1.50; 95% CI, 1.33-1.68) compared with the control group. For attainment of 2 or more primary targets, larger effects were observed among patients in low- and middle-income countries (OR, 1.50; 95% CI, 1.29-1.74) compared with high-income countries (OR, 1.20; 95% CI, 1.03-1.39) (*P* = .04).

**Conclusions and Relevance:**

In this 12-month clinical trial, the use of information and communications technology and nurses to empower and engage patients did not change the number of clinical events but did reduce cardiometabolic risk factors among patients with type 2 diabetes, especially those in low- and middle-income countries in the Asia-Pacific region.

**Trial Registration:**

ClinicalTrials.gov Identifier: NCT01631084

## Key Points

**Question:**

What are the effects of a quality improvement intervention on the care and cardiometabolic risk factors of patients with type 2 diabetes in low- and middle-income countries in the Asia-Pacific region?

**Findings:**

In this randomized clinical trial of 20 834 patients with type 2 diabetes in 8 Asia-Pacific countries, the intervention group received a technology-guided structured evaluation, automated personalized reports to encourage patient empowerment, and nurse reminders to increase patient engagement over a 12-month period. Clinical events were similar between the control and intervention groups at 12 months; however, the intervention group was more likely to experience reductions in multiple risk factors and increases in the attainment of diabetes-associated targets.

**Meaning:**

The study’s findings indicate that the use of information and communications technology and nurses to empower and engage patients did not change the number of clinical events but did reduce cardiometabolic risk factors among patients with type 2 diabetes in low- and middle-income countries.

## Teaser

This randomized clinical trial examines the effects of a quality improvement intervention comprising information and communications technology and increased contact with nurses on the clinical care and cardiometabolic risk factors of patients with type 2 diabetes in Asia-Pacific countries.

## Introduction

Large patient volume, multiple clinical needs, and insufficient patient engagement are major barriers to translating evidence into practice among patients with type 2 diabetes who require education and continuing care.^1,2^ Lack of capacity is an additional challenge in low- and middle-income countries.^3^ The phenotypic heterogeneity and silent nature of type 2 diabetes can lead to delayed intervention and treatment, patient treatment nonadherence, and worse outcomes, all of which are preventable. In a meta-analysis of quality improvement interventions involving 135 112 patients with type 2 diabetes, the use of team-based care, nonphysician personnel, and information and communications technology to improve patient-practitioner communication and patient self-management skills were associated with the largest effect sizes for the reduction of cardiometabolic risk factors.^4^

One in 5 patients with type 2 diabetes has multiple comorbidities (eg, myocardial infarction and stroke), and these comorbidities increase the risk of cardiovascular events and death by 2-fold to 7-fold in a dose-dependent manner.^5,6^ Although these adverse events can be ameliorated or prevented through early detection and control of multiple risk factors,^7^ substantial care gaps exist.^2,8,9^ In high-income countries, 1 in 4 patients with type 2 diabetes is able to attain all primary diabetes-associated targets, which are conventionally defined as a glycated hemoglobin A_1c_ (HbA_1c_) level lower than 7.0% (based on measurement standards from the National Glycohemoglobin Standardization Program^10^), a blood pressure (BP) level lower than 130/80 mm Hg, and a low-density lipoprotein (LDL) cholesterol level lower than 100 mg/dL (to convert to millimoles per liter, multiply by 0.0259).^6,8^ In addition, 40% to 60% of patients with type 2 diabetes in high-income countries receive organ-protective drugs.^6,8^ In Asia, which comprises many low- and middle-income countries, the proportion of patients with type 2 diabetes meeting all primary diabetes-associated targets has been reported to be lower than 10%, and only 30% to 40% of patients have received organ-protective drugs.^11^

More than 60% of patients with type 2 diabetes live in Asia, with the largest populations in China and India.^12^ In these low- and middle-income countries, health care systems are often unprepared to address the increasing number of patients with type 2 diabetes, which means that many patients with type 2 diabetes are not assessed or treated, and their disease is not adequately controlled.^9,13^ In high-income countries, quality improvement interventions using nonphysician personnel (eg, nurses, pharmacists, dietitians, community health care workers or peer supporters, and expert patients) and information and communications technology to complement medical care have been associated with improvements in risk factors and self-management skills and reductions in diabetes-associated complications.^14,15,16^

In 1995, we designed the Hong Kong Diabetes Register through modification of clinical setting, workflow, and team structure and used the data collected to stratify risk, empower patients, and help physicians identify care gaps.^9^ As part of health care policy reform in 2000, this care model was translated into a territory-wide risk assessment and management program, which helped to reduce mortality rates by 50% to 70% among patients with type 2 diabetes between 2001 and 2016.^16^ We used data from the Hong Kong Diabetes Register to develop equations and risk categories to estimate complications. In 2007, we developed the Joint Asia Diabetes Evaluation (JADE) technology, consisting of a web-based multifunctional multilingual portal that incorporated protocols and risk engines to guide data collection.^17^ The data formed the basis of clinic-based registers for quality assurance and were used to issue the JADE personalized reports with the aim of empowering patients and promoting shared decision-making.^17^

Few quality improvement interventions are available for patients with type 2 diabetes in Asia despite the substantial disease burden. We hypothesized that the use of trained nurses, who were supervised by physicians and guided by the JADE technology to evaluate, empower, and engage patients, would reduce diabetes-associated complications and improve cardiometabolic risk factors among patients with type 2 diabetes in the Asia-Pacific region.

## Methods

### Study Design

The JADE program was designed, implemented, and coordinated (with ongoing evaluation) by the Asia Diabetes Foundation (ADF), a nonprofit research organization governed by the Chinese University of Hong Kong Foundation. From June 28, 2012, to April 28, 2016, we conducted a 12-month randomized clinical trial of a quality improvement intervention at 50 sites across 8 countries in Asia (India, Indonesia, Malaysia, the Philippines, Singapore, Taiwan, Thailand, and Vietnam). The study protocol was approved by the joint research ethics committee of the Chinese University of Hong Kong–New Territories East Cluster Clinical Research Ethics Committee and local institutional review boards (the trial protocol is available in Supplement 1). All patients provided written informed consent before enrollment. This study followed the Consolidated Standards of Reporting Trials (CONSORT) reporting guideline for randomized clinical trials.

Adult patients (aged ≥18 years) with type 2 diabetes who were receiving treatment with lifestyle modification programs and/or any glucose-lowering drugs, including insulin, were eligible. Patients with type 1 diabetes (defined as acute presentation with diabetic ketoacidosis, heavy ketonuria [blood ketone level >3], or continuous insulin requirements within 1 year of diagnosis), mental disorders, and life-threatening illnesses were excluded. The first patient visit occurred in June 2012, and the last patient visit occurred in April 2016.

### Patients and Procedures

The JADE technology consists of a web-based portal that contains templates for guiding structured evaluation workflow, clinical decision support, and evidence-based care protocol.^9,17^ The JADE portal facilitates the structured collection of clinical and biochemical data to stratify patients into 1 of 4 distinct risk categories (from low risk to very high risk) for estimating cardiovascular-kidney events and all-cause mortality.^9^ The JADE report (eFigure 1 in Supplement 2) displays the 5-year probability of first cardiovascular-kidney events and patterns or targets of risk factors, with tailored recommendations for patients (eg, self-management skills and treatment adherence) and health care professionals (eg, treatment intensification, referral for education, and follow-up schedule). The JADE reports are available in 8 languages (English, traditional Chinese, simplified Chinese, Indonesian, Korean, Malay, Thai, and Vietnamese).

All participating physicians and nurses attended a 1½-day training workshop conducted by the ADF, during which they were informed about the study objectives and design. The training workshop included lectures, demonstrations, and role-play exercises. Physicians were advised to provide a dedicated workspace, located away from the busiest sections of their clinics, that ensured access to basic tools and office equipment for the nurses to implement the quality improvement intervention. By changing the setting in which the intervention occurred, nurses were in a better position to engage patients.

Patients were first scheduled for a structured evaluation session, in which the nurses used structured care report forms to collect relevant information (eg, sociodemographic characteristics, medication receipt, and lifestyle factors), perform physical assessments (eg, BP levels, anthropometric measurements, and 12-lead electrocardiographic examinations), conduct foot and eye examinations (eg, 10-g monofilament testing, graduated tuning fork testing, and retinal photography, if available), and collect blood and urine samples. If uncertain about any aspect of the evaluation process, the nurses could seek medical support to complete the clinical details. Physicians were encouraged to record important information on medical history and physical examination findings (eg, results of foot pulse and eye examinations if Doppler scans or referrals to ophthalmologists were not routine practices).

Nurses entered all anonymized data into the JADE portal, issued the personalized reports, explained the reports to patients, and returned telephone calls to patients as needed. Both physicians and nurses were trained to interpret and provide individualized explanations of the JADE report to empower patients and facilitate early intervention. Nurses could contact the physicians for any problems requiring medical attention that were detected during these sessions.

Each site was given access to the JADE portal to guide nurses in performing structured evaluations, using the JADE report to empower patients, and engaging patients with 2 or more telephone reminders or face-to-face contacts. For the nurse engagement component, nurses provided feedback to patients regarding their levels of risk factor control, encouraged patients to clarify uncertain areas (eg, lifestyle, medications, and emotions), empowered patients with skills for self-management, and provided psychological support to help patients develop the confidence to attain multiple treatment targets. After overnight fasting, all patients received a structured evaluation at baseline and at 12 months in addition to usual care.

### Randomization and Blinding

The study comprised 2 phases; phase 1 was conducted from June 2012 to August 2015, and phase 2 was conducted from June 2012 to April 2016. In both phases, the intervention group received 3 care components: a nurse-led JADE technology–guided structured evaluation, automated personalized reports to encourage patient empowerment, and 2 or more telephone reminders or face-to-face contacts by nurses to increase patient engagement. During phase 1, the control group received the JADE technology–guided structured evaluation and automated personalized reports. During phase 2, the control group received the JADE technology–guided structured evaluation only.

**Figure 1.**
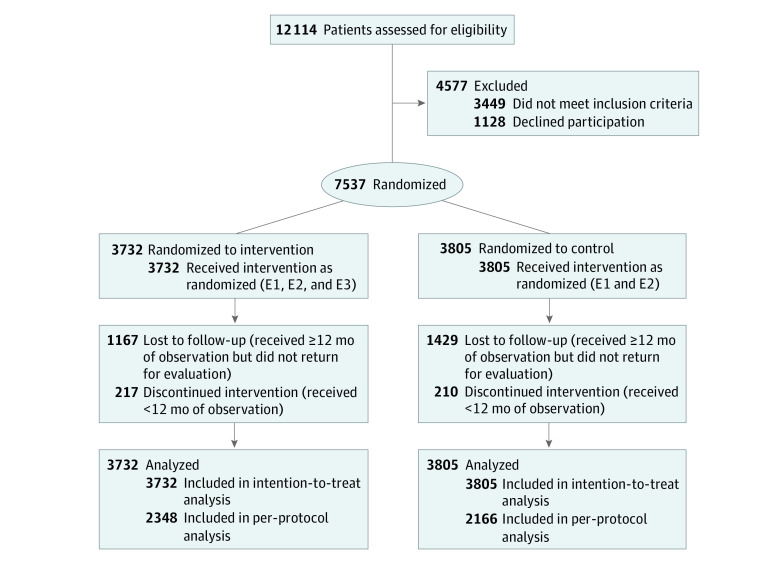
CONSORT Diagram for Phase 1 Study E1 indicates structured evaluation (standardized data collection, physical assessment, foot and eye examinations, and laboratory measurements) guided by web-based Joint Asia Diabetes Evaluation technology; E2, automated personalized reports to encourage patient empowerment; and E3, 2 or more telephone or face-to-face contacts by nurses to increase patient engagement over a 12-month period.

In phase 1, patients from 6 countries (India, Malaysia, Singapore, Taiwan, Thailand, and Vietnam) were randomized to either the intervention or control group on a 1:1 ratio using computer-generated codes kept in sealed and opaque envelopes, which were prepared by the ADF for each site. An independent on-site staff member opened the envelopes and informed participants of their group assignment. Given the study’s pragmatic design, participating patients, physicians, and nurses were not blinded to patients’ group assignments (CONSORT diagram for phase 1 is shown in Figure 1).

**Figure 2.**
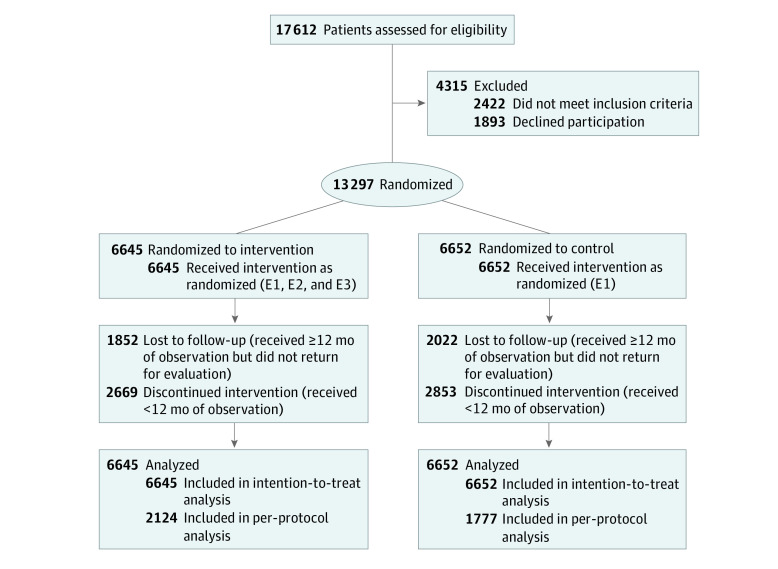
CONSORT Diagram for Phase 2 Study E1 indicates structured evaluation (standardized data collection, physical assessment, foot and eye examinations, and laboratory measurements) guided by web-based Joint Asia Diabetes Evaluation technology; E2, automated personalized reports to encourage patient empowerment; and E3, 2 or more telephone or face-to-face contacts by nurses to increase patient engagement over a 12-month period.

During the performance of the study, results from similar studies in Hong Kong^18,19^ and China^20^ emerged, indicating that the personalized JADE report was associated with substantial improvements in risk factors and attenuation of between-group differences. Therefore, in phase 2, patients from 4 countries (India, Indonesia, Malaysia, and the Philippines) who were randomized to the control group received technology-guided structured evaluations but did not receive personalized JADE reports (CONSORT diagram for phase 2 is shown in Figure 2).

To optimize implementation fidelity, the ADF conducted online site monitoring using the JADE portal. After the training workshop, each site was given an operating manual with step-by-step instructions on evaluating patients, using the JADE portal to collect data and issue personalized reports, and explaining the personalized JADE reports to patients. The manual also contained questions frequently asked by patients, with sample answers for physicians and nurses to reference. To keep the study teams engaged, the ADF issued progress reports and project newsletters every 3 to 6 months, which included information on recruitment status and data completeness by site and country and provided reminders regarding all study procedures.

### Outcomes

The primary outcome was the incidence of a composite of diabetes-associated end points, including cardiovascular disease, chronic kidney disease (defined as an estimated glomerular filtration rate of <60 mL/min/1.73 m^2^) or end-stage kidney disease (defined as the receipt of dialysis or an estimated glomerular filtration rate of <15 mL/min/1.73 m^2^), visual impairment or eye surgery, lower extremity amputation or foot ulcers requiring hospitalization, all-site cancers, and death. Secondary outcomes were the attainment of 2 or more primary diabetes-associated targets (HbA_1c_ <7.0%, BP <130/80 mm Hg, and LDL cholesterol <100 mg/dL) and/or the attainment of 2 or more key performance indices (reduction in HbA_1c_ ≥0.5%, reduction in systolic BP ≥5 mm Hg, reduction in LDL cholesterol ≥19 mg/dL, and reduction in body weight ≥3.0%). Sustained reduction of these cardiometabolic risk factors has been associated with decreases in the risk of cardiovascular-kidney events and death.^21^ Patients who did not have a 12-month second evaluation by 3 months before or after their prespecified date were considered to be lost to follow-up.

### Sample Size Calculation

In a previous 12-month pilot project, we randomized, on a 1:1 ratio, 240 patients with type 2 diabetes who had a history of ischemic heart disease or multiple risk factors. Patients received a JADE-assisted evaluation followed by allocation to either a control group, which received usual care, or an intervention group, which received additional nurse visits every 2 to 3 months at the Prince of Wales Hospital Diabetes Centre in Hong Kong.^22^ The proportion of patients in the intervention group who attained 2 or more primary targets increased 2.5-fold (from 15% at baseline to 37% at 12 months) compared with patients in the control group, for which the proportion increased 1.8-fold (from 15% at baseline to 27% at 12 months). Consistent with these findings, another multicenter randomized clinical trial (Effects of Structured vs Usual Care on Renal Endpoint in Type 2 Diabetes [SURE]) involving 205 patients with type 2 diabetes and chronic kidney disease reported that structured care delivered by a physician-nurse team improved the attainment of multiple targets by 2-fold (61%) compared with usual care (28%).^23^

A review of the literature indicated that 12% to 36% of patients with type 2 diabetes attained 2 or more primary targets.^11,24,25^ Based on previous studies,^11,24,25^ we anticipated that treatment target attainment would increase by 50% in the control group and 2-fold in the intervention group. Assuming a default rate of 20%, a sample of 295 to 1814 patients per site would provide 80% power at a 5% significance level to confirm the benefits of the JADE-assisted multicomponent care in attaining 2 or more primary targets, depending on baseline proportions. Based on our experience, a nurse is generally able to evaluate 4 patients every morning. With access to basic facilities (eg, consultation room, telephone, and assessment tools) and physician supervision, we estimated that a full-time nurse could enroll 600 patients, perform 2 technology-guided evaluations per patient at baseline and 12 months, and provide additional patient support via face-to-face or telephone consultations (1 hour per patient) in the intervention group (n = 300). We therefore provided each site with a grant equivalent to a nurse’s salary for 18 months to facilitate implementation of the quality improvement intervention. We overrecruited participants to provide sufficient power for each site to perform its own analysis after the primary analysis was completed.

### Statistical Analysis

We conducted both intention-to-treat and per-protocol analyses. All randomized patients were included in the intention-to-treat analysis. Patients who received at least 12 months of observation and returned for a follow-up evaluation at 12 months were included in the per-protocol analysis. Multiple imputations were performed using the predictive mean matching method for handling missing data.^26^ All data were expressed as means with SDs, medians with interquartile ranges (IQRs), and numbers with percentages, as appropriate. For between-group comparisons, we used χ^2^ tests or Fisher exact tests for categorical variables and unpaired independent *t* tests or Wilcoxon rank sum tests for continuous variables.

Logistic regression analyses, adjusted for country, were used to examine the effects of the intervention on primary and secondary outcomes compared with the control conditions. Patients with a documented history of any event of interest were excluded from the logistic regression models assessing the primary outcome. For secondary outcomes, all randomized patients were included in the logistic regression models. The adjusted effect estimates were presented as odds ratios (ORs) with 95% CIs. The phase 1 study included sites from low-, middle-, and high-income countries (based on 2019 World Bank income categories^27^), and the phase 2 study included sites from low- and middle-income countries only. In the phase 1 study, we tested interaction between the intervention group (yes or no) and low- and middle-income countries (yes or no) using logistic regression models followed by stratified analyses. All analyses were performed using R software, version 3.6.3 (R Foundation for Statistical Computing), with a 2-tailed significance threshold of *P* < .05. Data were analyzed from July 3, 2019, to July 21, 2020.

## Results

### Study Population

From 2012 to 2015, 20 834 patients with type 2 diabetes were recruited in both phases. In phase 1, 7537 patients were randomized, with 3732 patients randomized to the intervention group and 3805 patients randomized to the control group (Figure 1). In phase 2, 13 297 patients were randomized, with 6645 patients randomized to the intervention group and 6652 patients randomized to the control group (Figure 2). The per-protocol analyses, which included patients with at least 12 months of observation who returned for a second evaluation at 12 months, comprised 4514 patients in phase 1 and 3901 patients in phase 2.

### Baseline Characteristics

The Table shows the baseline characteristics of all randomized patients in the phase 1 and phase 2 studies. Among 7537 randomized participants in phase 1, the mean (SD) age was 60.0 (11.3) years, 3914 patients (51.9%) were male, 4855 patients (64.4%) were from low- and middle-income countries, the median duration of type 2 diabetes was 10 years (IQR, 5-15 years), and the mean (SD) HbA_1c_ was 7.85% (1.71%). A total of 1177 patients (15.6%) had cardiovascular disease, and 1322 patients (17.5%) had chronic kidney disease; 3816 patients (50.6%) were receiving statin medications, and 3292 patients (43.7%) were receiving renin-angiotensin system inhibitors. In total, 2405 patients (31.9%) had already attained 2 or more primary targets at baseline.

**Table.** Baseline Characteristics of Participants in Phase 1 and Phase 2 Studies

Characteristic	No. (%)			
	Phase 1 (n = 7537)^a^		Phase 2 (n = 13 297)^b^	
	Intervention group	Control group	Intervention group	Control group
Total participants, No.	3732	3805	6645	6652
Age, mean (SD), y	60.0 (11.2)	59.9 (11.4)	54.0 (11.2)	54.2 (11.2)
Duration of diabetes, median (IQR), y	9 (5-15)	10 (5-15)	7 (3-13)	7 (3-13)
Male sex	1946 (52.1)	1968 (51.7)	3869 (58.2)	3885 (58.4)
Female sex	1786 (47.9)	1837 (48.3)	2776 (41.8)	2767 (41.6)
National income level^c^				
High	1339 (35.9)	1343 (35.3)	0	0
Upper middle	599 (16.1)	594 (15.6)	250 (3.8)	283 (4.3)
Lower middle	1794 (48.1)	1868 (49.1)	6395 (96.2)	6369 (95.7)
≥College educational level	1509 (40.4)	1405 (36.9)	3244 (48.8)	3226 (48.5)
Current smoker	336 (9.0)	365 (9.6)	631 (9.5)	615 (9.2)
HbA_1c_, mean (SD), %^d^	7.81 (1.67)	7.86 (1.74)	8.44 (1.89)	8.37 (1.86)
BP, mean (SD), mm Hg				
Systolic	133.6 (17.2)	133.4 (16.9)	129.9 (15.5)	130.0 (15.7)
Diastolic	79.1 (9.2)	78.9 (9.4)	80.1 (8.5)	80.1 (8.5)
Cholesterol, mean (SD), mg/dL				
Total	173.63 (42.54)	174.40 (42.92)	179.04 (47.56)	179.82 (43.70)
LDL	97.45 (39.06)	99.00 (40.60)	102.09 (41.76)	104.02 (46.02)
HDL	44.86 (13.92)	44.86 (15.47)	42.92 (15.08)	43.31 (15.85)
Triglycerides, median (IQR), mg/dL	133.64 (95.58-187.62)	132.75 (94.70-186.74)	146.03 (110.63-190.28)	145.14 (109.74-190.28)
BMI, mean (SD)	26.4 (4.5)	26.4 (4.7)	27.5 (4.8)	27.4 (4.9)
Estimated GFR, mean (SD), mL/min/1.73 m^2^	81.0 (22.9)	81.4 (23.3)	83.5 (22.4)	83.4 (22.4)
Chronic kidney disease	650 (17.4)	672 (17.7)	809 (12.2)	822 (12.4)
Cardiovascular disease	591 (15.8)	586 (15.4)	898 (13.5)	829 (12.5)
Medications				
Renin-angiotensin system inhibitors	1633 (43.8)	1659 (43.6)	1641 (24.7)	1686 (25.3)
Statins	1929 (51.7)	1887 (49.6)	2004 (30.2)	1983 (29.8)
Oral glucose-lowering drugs	3118 (83.5)	3169 (83.3)	5886 (88.6)	5836 (87.7)
Insulin	919 (24.6)	1019 (26.8)	1869 (28.1)	1742 (26.2)
Primary targets				
HbA_1c_ <7.0%	1277 (34.2)	1284 (33.7)	1325 (19.9)	1367 (20.6)
BP <130/80 mm Hg	867 (23.2)	895 (23.5)	1352 (20.3)	1381 (20.8)
LDL cholesterol <100 mg/dL	2100 (56.3)	2090 (54.9)	3184 (47.9)	3077 (46.3)
≥2 Primary targets attained	1222 (32.7)	1183 (31.1)	1396 (21.0)	1400 (21.0)

Abbreviations: BMI, body mass index (calculated as weight in kilograms divided by height in meters squared); BP, blood pressure; GFR, glomerular filtration rate; HbA_1c_, glycated hemoglobin A_1c_; HDL, high-density lipoprotein; IQR, interquartile range; JADE, Joint Asia Diabetes Evaluation; LDL, low-density lipoprotein.

SI conversion factors: To convert LDL cholesterol to millimoles per liter, multiply by 0.0259; and triglycerides to millimoles per liter, multiply by 0.0113.

^a^ Phase 1 was conducted from June 2012 to August 2015 in 6 countries (India, Malaysia, Singapore, Taiwan, Thailand, and Vietnam). The intervention group received structured evaluation guided by web-based JADE technology, automated personalized reports for empowerment, and 2 or more telephone or face-to-face contacts by nurses over a 12-month period. The control group received structured evaluation guided by web-based JADE technology and automated personalized reports for empowerment.

^b^ Phase 2 was conducted from June 2012 to April 2016 in 4 countries (India, Indonesia, Malaysia, and the Philippines). The intervention group received structured evaluation guided by web-based JADE technology, automated personalized reports for empowerment, and 2 or more telephone or face-to-face contacts by nurses over a 12-month period. The control group received structured evaluation guided by web-based JADE technology only.

^c^ Based on 2019 World Bank income categories.^27^

^d^ Based on measurement standards from the National Glycohemoglobin Standardization Program.^10^

Among 13 297 participants randomized in phase 2, the mean (SD) age was 54.0 (11.1) years, 7754 patients (58.3%) were male, 13 297 patients (100%) were from low- and middle-income countries, the median duration of type 2 diabetes was 7 years (IQR, 3-13 years), and the mean (SD) HbA_1c_ was 8.39% (1.88%). A total of 3987 patients (30.0%) were receiving statin medications, and 3327 patients (25.0%) were receiving renin-angiotensin system inhibitors. Only 2796 patients (21.0%) had already attained 2 or more primary targets at baseline.

### Outcomes

**Figure 3.**
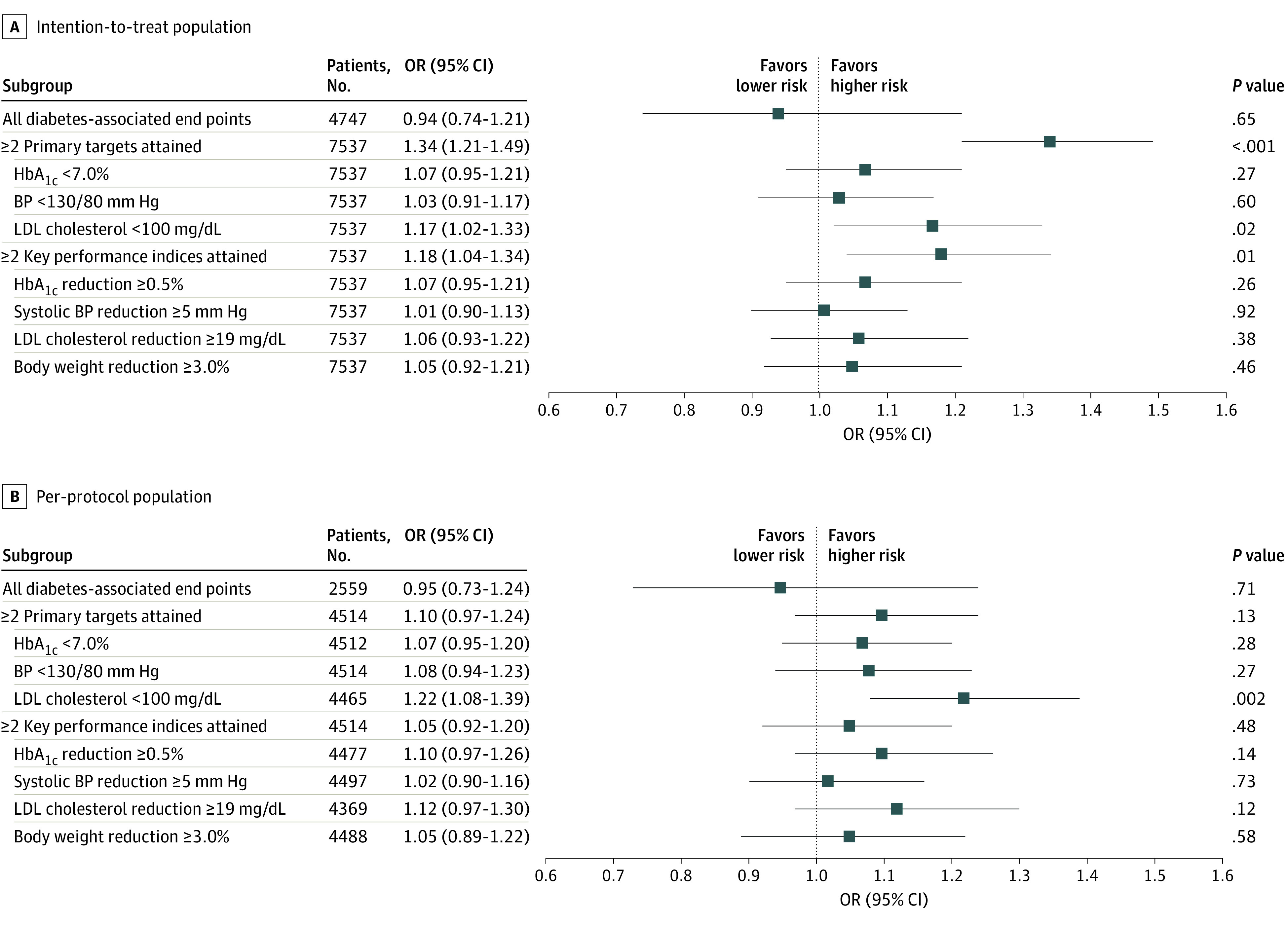
Effects of Intervention vs Control Conditions on Cardiometabolic Risk Factors Among Participants in Phase 1 Study To convert low-density lipoprotein (LDL) cholesterol from milligrams per deciliter to millimoles per liter, multiply by 0.0259. HbA_1c_ indicates glycated hemoglobin A_1c_; BP, blood pressure; OR, odds ratio.

In the phase 1 study, at 12 months, the risk of experiencing any of the diabetes-associated end points was similar between the intervention and control groups in both the intention-to-treat (OR, 0.94; 95% CI, 0.74-1.21) and per-protocol (OR, 0.95; 95% CI, 0.73-1.24) analyses after adjusting for country (Figure 3). In the intention-to-treat analysis, the intervention group had an increased likelihood of attaining 2 or more primary targets (OR, 1.34; 95% CI, 1.21-1.49) and 2 or more key performance indices (OR, 1.18; 95% CI, 1.04-1.34) compared with the control group (eTable 1 in Supplement 2). Although similar, the effects were not statistically significant in the per-protocol analysis. Among the primary targets, the effect of the intervention on LDL cholesterol levels (ie, reducing LDL cholesterol <100 mg/dL) was consistent in both the intention-to-treat (OR, 1.17; 95% CI, 1.02-1.33) and per-protocol (OR, 1.22; 95% CI, 1.08-1.39) analyses (Figure 3). When stratified by national income level, compared with high-income countries, low- and middle-income countries had an increased likelihood of attaining 2 or more primary targets (OR, 1.50 [95% CI, 1.29-1.74] vs 1.20 [95% CI, 1.03-1.39], respectively; *P* = .04) and 2 or more key performance indices (OR, 1.62 [95% CI, 1.35-1.94] vs 0.86 [95% CI, 0.72-1.03], respectively; *P* < .001) (eFigure 2 in Supplement 2).

**Figure 4.**
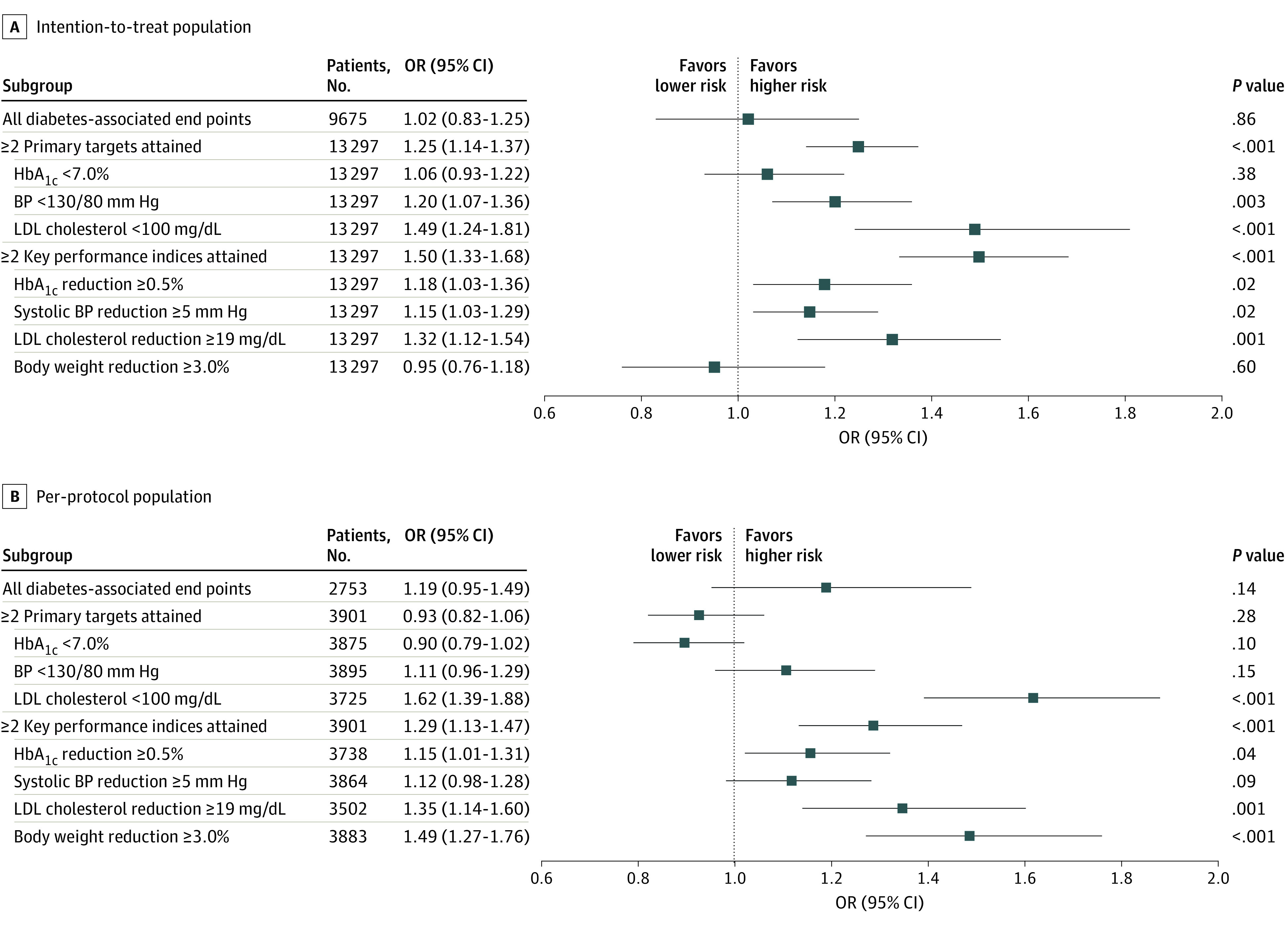
Effects of Intervention vs Control Conditions on Cardiometabolic Risk Factors Among Participants in Phase 2 Study To convert low-density lipoprotein (LDL) cholesterol from milligrams per deciliter to millimoles per liter, multiply by 0.0259. HbA_1c_ indicates glycated hemoglobin A_1c_; BP, blood pressure; OR, odds ratio.

In the phase 2 study, the risk of experiencing any of the diabetes-associated end points was similar between the intervention and control groups in the intention-to-treat analysis (OR, 1.02; 95% CI, 0.83-1.25) (Figure 4). In the intention-to-treat analysis, the intervention group had an increased likelihood of attaining 2 or more primary targets (OR, 1.25; 95% CI, 1.14-1.37) and 2 or more key performance indices (OR, 1.50; 95% CI, 1.33-1.68) compared with the control group (eTable 1 in Supplement 2). In the per-protocol analysis, the effect of the intervention on the attainment of 2 or more key performance indices remained significant (OR, 1.29; 95% CI, 1.13-1.47) but was not significant for the attainment of 2 or more primary targets (OR, 0.93; 95% CI, 0.82-1.06). Both the intention-to-treat and per-protocol analyses showed consistent results for the primary target of attaining an LDL cholesterol level of below 100 mg/dL and the key performance indices of reducing HbA_1c_ level by 0.5% or more and reducing LDL cholesterol level by 19 mg/dL or more (Figure 4).

Among those who had at least 12 months of observation, 2596 patients (34.4%) in phase 1 and 3874 patients (29.1%) in phase 2 did not return for a structured evaluation. A total of 4514 patients (59.9%) in phase 1 and 3901 patients (29.3%) in phase 2 returned for a structured evaluation after at least 12 months of observation. In the phase 1 study, patients who returned for evaluation were older, had longer disease duration, had greater frequency of a history of cardiovascular disease or chronic kidney disease, had higher medication receipt (with the exception of insulin), and had higher target attainment at baseline compared with patients who did not return for evaluation (eTable 2 in Supplement 2). Similar characteristics were observed among participants in the phase 2 study (eTable 3 in Supplement 2).

## Discussion

In this 12-month multinational randomized clinical trial of a quality improvement intervention, a multicomponent program using trained nurses under physician supervision and assistance from the JADE technology to evaluate, empower, and engage patients did not alter the number of clinical events among Asian patients with type 2 diabetes compared with the control group. However, the intervention group was more likely to attain multiple treatment targets and experienced clinically meaningful reductions in cardiometabolic risk factors, with greater effect sizes among participants in low- and middle-income countries compared with high-income countries. Despite the diverse care settings, results from this study support the use of information and communications technology and nonphysician personnel to decrease care gaps among patients with type 2 diabetes. This quality improvement intervention focused on the transfer of knowledge to promote self-management skills, treatment adherence, and improvements in ongoing care. Nurses provided an important means of relaying information between physicians and patients to ensure data were collected, documented, and communicated to promote shared decision-making.

Because of the relatively brief follow-up period, we were not able to demonstrate differences in clinical events because the effects of multiple risk factor reduction will likely take years to emerge.^28^ In Hong Kong, after a median follow-up of 4.5 to 6.0 years, the implementation of a similar risk assessment and management program complemented by a patient empowerment program in primary and secondary care settings was associated with a 30% to 60% reduction in the risk of cardiovascular-kidney events and death.^17,29^ In previous randomized clinical trials (such as the Intensified Multifactorial Intervention in Patients With Type 2 Diabetes and Microalbuminuria [Steno-2] study, the Japan Diabetes Optimal Integrated Treatment Study for 3 Major Risk Factors of Cardiovascular Diseases [J-DOIT3], and the SURE study, which were characterized by the use of protocols, care coordinators, standardized data collection, and access to essential medications), marked improvement in clinical outcomes was observed, even among participants in the control groups.^7,23,30^ In the present study, by training nurses to perform the JADE technology–guided structured evaluations, we observed reductions in multiple risk factors after 12 months, supporting the notion that the provision of structured care using information and communications technology and nonphysician personnel can facilitate systematic data collection to change actions in care delivery.^31^ By using personalized reports to explain these data to patients together with nurse reminders to empower and engage patients, this quality improvement intervention resulted in a greater likelihood of clinically meaningful reductions in systolic BP (≥5 mm Hg), HbA_1c_ (≥0.5%), LDL cholesterol (≥19 mg/dL), and/or body weight (≥3.0%) among the intervention group. These improvements, if sustained, may lead to reductions in cardiovascular-kidney events and death.

In the phase 1 study, all patients received the personalized JADE reports that were written in everyday language. This empowerment component might have attenuated the benefits of the additional nurse contacts, which were designed to engage patients and reinforce self-management skills. In the phase 2 study, the control condition represented a more accurate real-world situation, in which patients are often not informed of the results of their evaluations, especially if they reside in low- and middle-income countries that have scarce health care resources and limited time available for patient-practitioner consultations. It was also in these low- and middle-income countries where the effect of our intervention was most evident. Because of the low physician-to-patient ratio in low- and middle-income countries,^32^ a heavy workload often produces care fragmentation, patient treatment nonadherence, and burnout among health care professionals.^33^ The present study attempted to reduce the heavy workload of physicians by providing funding to hire a nurse and the JADE technology with built-in protocols and decision support, which could be used to train nursing staff to complement medical care. However, because of the high default rate, brief follow-up duration, overestimation of effect size, and other unforeseen factors, we were not able to confirm the primary outcome of clinical events. These unforeseen factors included staffing changes, insufficient capacity, lack of financial coverage for structured evaluations and medications, lack of patient literacy and readiness to receive care from nonphysician personnel, and lack of institutional support. Despite these challenges, the reduction of multiple risk factors supported our hypothesis that the use of nonphysician personnel, guided by information and communications technology, was able to decrease care gaps, especially in low- and middle-income countries. Thus, by changing the setting and empowering nurses to deliver the JADE-assisted program and facilitate communication among all parties concerned, we could start to build capacity and empower not only the patients but the health care team to deliver evidence-based integrated care.

The analyses of low-, middle-, and high-income countries were performed post hoc. The lack of infrastructure and capacity in low- and middle-income countries might have resulted in greater benefits for facilities in those countries compared with high-income countries, as the additional manpower and technology provided the necessary resources to help sites in low- and middle-income countries evaluate, empower, and engage patients. Better practice environments in high-income countries vs low- and middle-income countries might also have attenuated the differences between the intervention and control groups. The seemingly counterintuitive results indicating that the intervention was associated with a lower, albeit not statistically significant, likelihood of reducing clinical events in high-income countries might be owing to ascertainment bias, as facilities in high-income countries are better able to document events and have greater availability of laboratory tests compared with facilities in low- and middle-income countries. Despite the potential of the JADE program, a conducive macroenvironment and team alignment are as important as patient engagement to overcoming the multiple behavioral and administrative barriers to delivering high-quality diabetes care.

### Strengths and Limitations

This study has strengths. The study was multinational and comprised a broad range of health care facilities, including hospital and community-based clinics with universal, subsidized, or private payment structures. This heterogeneity means that the study’s results are relevant to many settings.

This study also has limitations. These limitations can inform future design and implementation of pragmatic research aimed at defining and evaluating operational strategies to create change in the care of patients with type 2 diabetes. First, by its nature, the study was not blinded, and the diversity of the health care systems included might have affected implementation fidelity and default rates. This limitation was minimized by the provision of training to all physician-nurse teams, the use of standardized protocols and case record forms, and the implementation of online monitoring by the ADF, with regular site progress reports and project newsletters.

Second, the 1-year default rate observed in this clinical trial of a quality improvement intervention was higher than that in most randomized clinical trials that receive funding for on-site monitoring and compensation to patients, health care professionals, and institutions. To that end, most randomized clinical trials provide evidence of efficacy, while clinical trials of real-world interventions provide evidence of effectiveness and identify barriers and enablers for translating evidence into practice. In many low- and middle-income countries, the costs associated with structured evaluations (especially laboratory tests) and medications are paid out of pocket and not covered by health care systems. In this quality improvement intervention, we did not cover the costs for assessments and medications. Depending on the extent of subsidies, the participants’ inability or unwillingness to pay might explain the approximately 30% default rate among those scheduled to return for evaluation at 12 months. A similar default rate was reported in the China JADE Program, suggesting that these defaults are real-world occurrences.^20^ Even with close monitoring and provision of incentives to retain patients, treatment discontinuation rates as high as 25% have been reported in pharmaceutical industry–sponsored clinical trials lasting several years.^34^ Despite these challenges, the risk factor reduction associated with our multicomponent intervention remained clinically substantial in both the intention-to-treat and per-protocol analyses.

Third, volunteer bias among both the patients and participating study teams could limit the generalizability of the study’s results. Fourth, qualitative research is needed to understand the perspectives of patients, health care professionals, and payers regarding the implementation, acceptance, and adherence to quality improvement interventions such as this one, which will enable further adaptation of these interventions to address local needs. Fifth, participants who did not return for evaluation were younger and had higher educational levels but lower target attainment than those who returned for evaluation. Low levels of risk awareness, competing life priorities, and/or demand for high-quality services among younger individuals, which may result from higher literacy, suggest a need for refinement to make this technology-enhanced team-based care model more user friendly and engaging.^31,35^

## Conclusions

Diabetes is a complex condition requiring multidimensional intervention to reduce multiple risk factors and prevent complications. By using a multicomponent approach that included nurse engagement as well as information and communications technology, the present intervention was able to improve the control of multiple cardiometabolic risk factors. Given the relatively low costs of the JADE program, which involved training nurses to perform annual structured evaluations guided by the JADE portal and to use personalized reports to empower patients and promote shared decision-making, this care model might provide a readily available solution, especially in low- and middle-income countries, to improve clinical outcomes among patients with type 2 diabetes pending other health care reforms.

## Notes

Supplement 1. **Trial Protocol**

Supplement 2. **eFigure 1.** Example of JADE Personalized Report **eFigure 2.** Effects of Intervention (E1+2+3) vs Control (E1+2) on Cardiometabolic Risk Factors in Phase 1 Study, Stratified by National Income Level (Intention-to-Treat and Per-Protocol Analyses) **eTable 1.** Changes in Medication Usage and the Proportion of Patients Attaining Treatment Targets and Key Performance Indices in the Intention-to-Treat Population **eTable 2.** Comparison of Baseline Characteristics Between Patients With at Least 12 Months of Observation Who Did and Did Not Return for Repeat Evaluation in Phase 1 Study **eTable 3.** Comparison of Baseline Characteristics Between Patients With at Least 12 Months of Observation Who Did and Did Not Return for Repeat Evaluation in Phase 2 Study

Supplement 3. **Data Sharing Statement**
