# Supplementary material for: Effects of a Technology-Assisted Integrated Diabetes Care Program on Cardiometabolic Risk Factors Among Patients With Type 2 Diabetes in the Asia-Pacific Region: The JADE Program Randomized Clinical Trial
Source: JAMA Netw Open. 2021 Apr 30;4(4):e217557. doi: 10.1001/jamanetworkopen.2021.7557 (PMC8087959; doi:10.1001/jamanetworkopen.2021.7557)
Supplement: Supplement 1. — Trial Protocol [file jamanetwopen-e217557-s001.pdf]

AP-JD Program

A Multicentre Randomized Program to Compare the Effect of the Joint Asia Diabetes Evaluation (JADE, structured care) versus the DIAbetes MONitoring Database (DIAMOND, usual care) Programs in Type 2 Diabetic Patients in Asia Pacific Region

Proposed by

Professor Juliana CN Chan (Principal Investigator)

on behalf of the

Asia Diabetes Foundation (ADF)

Chinese University of Hong Kong - Prince of Wales Hospital

- International Diabetes Federation Centre of Education (CUHK-PWH-IDFCE)

Hong Kong Project Team (in alphabetical order)

Ms. Nicola Brown, Research Associate, ADF
Dr. Chun-Chung Chow, Consultant, PWH
Ms. Harriet Chung, Nurse Specialist, CUHK
Dr. Andrea Luk, Resident Specialist, PWH
Dr. Vanessa Ng, Resident Specialist, PWH
Dr. Risa Ozaki, Associate Consultant, PWH
Ms. Rebecca Wong, Nurse Consultant, PWH
Dr. Sui Yi, Research Associate, ADF
Ms. Jenny Zhang, PhD student, CUHK

Asia Diabetes Foundation

Table of Content

EXECUTIVE SUMMARY: ASIA PACIFIC – JD PROGRAM (WHAT, WHY & HOW) 3
What is the JADE Program? 3
Why do we need the JADE Program? 3
How to implement the JADE-DIAMOND (JD) Program? 4
Objectives and expected outcomes of AP -JD Program 4
ABSTRACT 5
BACKGROUND 6
Type 2 diabetes in Asia 6
Structured care 6
JADE Program 7
HYPOTHESIS 9
OBJECTIVES 9
STUDY DESIGN 10
SETTING AND PATIENTS 10
Inclusion criteria 11
Exclusion criteria 11
OUTCOME MEASURES 11
Composite primary endpoint (CRF (1-5)) 11
Composite secondary endpoint 12
Composite tertiary endpoint 12
STUDY PROTOCOL 13
Infrastructure, logistics and manpower 13
Randomization 13
Enrolment 14
Comprehensive assessment 14
Intervention – JADE Group 15
Intervention - DIAMOND group 18
Manpower estimation 18
SAMPLE SIZE CALCULATION 19
DATA ANALYSIS 25
DEFAULT AND MISSING DATA 26
SITE VISITS AND EVENT ADJUDICATION 26
BENEFITS AND SIGNIFICANCE 26
INTERLECTUAL PROPERTY 28
ABOUT ASIA DIABETES FOUNDATION 28
REFERENCES 30

EXECUTIVE SUMMARY: ASIA PACIFIC – JD PROGRAM (WHAT, WHY & HOW)

Diabetes is a global epidemic affecting 5-10% of the world population with China and India having the highest number of affected individuals. Diabetes reduces life expectancy by 6-12 years, if not well managed. It is the leading cause of stroke, heart disease, leg amputation, blindness and renal failure in many countries, which account for 50-60% of premature mortality and morbidity. Besides, there is increasing data suggesting close associations of diabetes with cancer and depression. Although controlling multiple risk factors improves clinical outcomes in type 2 diabetic patients, there are multiple challenges in translating evidence to clinical practice due to factors pertinent to patients, care providers and systems.

What is the JADE Program?

Since mid 1990s, the CUHK Diabetes Care & Research Group and others have advocated the use of a team comprising a doctor, nurse and health care assistant (HCA) to deliver protocol-augmented collaborative care. The key components of this collaborative care model include risk stratification, periodic assessments, reinforcement of compliance and control of multiple risk factors. These prototypes substantially improve rates of treatment compliance and attainment of multiple treatment targets resulting in reduced risks of death and cardiovascular-renal complications.

In 2007, supported by an educational grant, a charitable organization named the Asia Diabetes Foundation (ADF) (www.adf.org.hk) was established under the Chinese University of Hong Kong (CUHK) Foundation. The mandate of ADF is to develop and implement the Joint Asia Diabetes Evaluation (JADE) Program, which comprises a web-based disease management program to enable doctors, nurses and care professionals to manage people with diabetes in an integrated manner and to establish a diabetes registry for quality assurance purpose.

Why do we need the JADE Program?

In line with the recommendation by the International Diabetes Federation, the JADE electronic portal (e-portal) provides a virtual platform to facilitate implementation of evidence-based clinical protocols with ongoing data collection, management and analysis. The JADE Program incorporates validated risk equations developed by the CUHK Diabetes Care & Research Group for risk stratification and triage into different care protocols with

decision supports. This data is communicated to patients and care providers in the forms of charts and time trends to motivate behavioral changes and encourage collaborative goal setting and attainment.

<u>How to implement the JADE-DIAMOND (JD) Program?</u>

By participating in the JADE Program, doctors will be able to create their own diabetes registry for quality assurance and benchmark their performance including adherence to procedures, tracking of default rates and attainment of treatment targets. However, effective implementation of the JADE Program requires changes in practice environment and additional manpower (e.g. a nurse) to collect clinical data systematically, maintain a database and remind patients and doctors to adhere to protocols. As the first step towards this quality improvement program, structured templates can be used to guide care professionals to collect data systematically during comprehensive assessment to form a DIAbetes MONitoring Database (DIAMOND) for early detection of risk factors and complications.

<u>Objectives and expected outcomes of AP -JD Program</u>

In this demonstration project (Asia Pacific JADE and DIAMOND Program, AP-JD in short) supported by ADF, patients will be recruited from different sites across Asia, with each site recruiting at least 600 type 2 diabetic patients. After explanation by trained doctors and nurses, and with written informed consent, patients will be randomized to either the JADE (n=300, structured care) or DIAMOND (n=300, usual care) group. All patients will undergo a comprehensive assessment (CA) at baseline and yearly thereafter. Patients in the JADE group will be further managed by a doctor-nurse-HCA team according to a protocol based on risk stratification with predefined follow up (FU) schedules together with shared information and decision support (i.e. structured collaborative care). The DIAMOND protocol involves only baseline and yearly CA without predefined FU schedules or feedback of information between CA visits (i.e. usual care). The primary composite endpoint is all-diabetes related clinical endpoints. The secondary composite endpoint is attainment of treatment goals and/or control of risk factors. The tertiary changes are behavioral changes, psychological well being and quality of life.

ABSTRACT

Rationale: Change of practice environment and an interdisciplinary team is needed to implement evidence-based diabetes care which requires risk stratification, periodic assessments, education and treatment to targets.

Hypothesis: The use of a web-based disease management program (Joint Asia Diabetes Evaluation (JADE) Program) delivered by a doctor-nurse-HCA team reduces the incidence of all diabetes-associated clinical endpoint and improves control of risk factors compared to usual care (DIAbetes MONitoring Database (DIAMOND) group) in type 2 diabetes.

Objective: To compare structured care using the JADE portal versus usual care using the DIAMOND portal on incidence of all-diabetes related events, physical and psychological health.

Study design: A multicentre, randomized, integrated disease management program

Setting and patients: 600 patients from each site will be randomized to the JADE group (n=300) or the DIAMOND group (n=300) and followed up for at least 24 months.

Intervention: All patients will undergo comprehensive assessment (CA) guided by the templates in the respective portal at baseline, month 12 and 24. The JADE group will be further managed by a doctor-nurse-HCA team guided by the JADE portal with risk stratification program and recommendation to different care protocols with predefined follow up (FU) schedules, information sharing and decision supports.

Outcome measures: Primary composite outcomes include all diabetes-related clinical events; secondary composite outcomes include control of risk factors and tertiary composite outcomes include behavioral changes, psychological health, quality of life and cost-effective analysis.

BACKGROUND

Type 2 diabetes in Asia

Diabetes is now a pandemic disease affecting 5-10% of global population. More than 60% of affected people will come from Asia and the number is expected to increase from 85 million in 2005 to 132 million in 2010 in Asia alone (1). On average, diabetes reduces life expectancy by 6-12 years, if not well managed (2; 3). While stroke, heart disease and kidney failure account for more than 50% of global deaths, 30-50% of patients with these conditions have diabetes as a major causal factor (4). In contrast to the West, the main increase in diabetes prevalence in Asia will occur in the young to middle aged population (5; 6). In 2005, it was estimated that 17 million of deaths were due to stroke and heart disease, of which 11 million occurred in Asia, affecting many young parents and economically active people (7).

Structured care

Despite their devastating nature, many diabetic complications can be prevented, controlled and managed effectively to preserve health, reduce disabilities and improve quality of life. However, there are multiple barriers in the implementation of quality diabetes care which include periodic evaluation of clinical and laboratory parameters as well as support for people with diabetes to adhere to long term medications and self care. Due to the silent nature of diabetes and associated complications as well as the complex nature of care protocols, clinical inertia and poor compliance are not uncommon. Apart from the need to build capacity and strengthen the health care system to provide integrated care, lack of incentives and reimbursement for outpatient procedures including therapeutic patient education are other barriers in delivering quality chronic care (8-12).

Thus, although treatment to targets for risk factors can substantially reduce the risk of diabetes associated complications (13-18) especially in a clinical trial setting (19), the challenge lies in translating this evidence to daily practice. Since mid-1990s, inspired by the benefits of structured care, made possible during the conduct of clinical trials (20; 21) and in line with the results from metaanalysis (22-24) and international recommendations including that by the International Diabetes Federation (25; 26), the CUHK Diabetes Care & Research Group has developed structured care prototypes including regular comprehensive assessments (CA) implemented by a doctor-nurse-HCA team. In agreement with international data, these prototypes substantially improved rates of treatment compliance and attainment of multiple

treatment targets resulting in reduced death, cardio-renal complication and hospitalization rates (27-33).

IDF Global Guideline for care delivery

International Diabetes Federation Global Guidelines
Recommendations for diabetes care delivery

Standard care

CD1 Offer care to all people with diabetes, with sensitivity to cultural wishes and desires.
CD2 Encourage a collaborative relationship, by actively involving the person with diabetes in the consultation, and creating opportunities for them to ask questions and express concerns. Ensure that issues important to the person with diabetes are addressed.
CD3 Offer annual surveillance of all aspects of diabetes control and complications to all people with Type 2 diabetes
CD4 Agree a care plan with each person with diabetes, review this annually or more often if appropriate and modify it according to changes in wishes, circumstances and medical findings.
CD5 Use protocol-driven diabetes care to deliver the care plan between annual reviews, at booked routine reviews.
CD6 Provide urgent access to diabetes health-care advice for unforeseen problems.
CD7 Organize care around the person with diabetes.
CD8 Use a multidisciplinary care team with specific diabetes expertise maintained by continuing professional education.
CD9 Ensure that each person with diabetes is recorded on a list of people with diabetes, to facilitate recall for annual complications surveillance.
CD10 Provide telephone contact between clinic visits.
CD11 Consider how people with diabetes, acting as expert patients, and knowing their limitations, together with local/regional/national associations, might be involved in supporting the care delivery of their local health-care team.
CD12 Use data gathered in routine care to support quality assurance and development activities.

Comprehensive care

CDC1 In general this would be as *Standard care*.
CDC2 The person with diabetes will have access to their own electronic medical record via secure technology from remote sites. They will be able to give permission for any health-care professional to access that record.
CDC3 Decision support systems might be available to the health-care professional and perhaps to the person with diabetes.

Minimal care

CDM1 Offer annual surveillance, agree care plans, deliver protocol-driven care, and ensure that each person with diabetes is recorded on a local list of people with diabetes, as for *Standard care*.
CDM2 Organize care around the person living with diabetes, using an appropriately trained health to deliver the diverse aspects of that care

JADE Program

The JADE Program is a web-based disease management program conceptualized, developed and tested by the ADF since 2007 supported by a MSD educational grant (34-36). The objectives of the JADE Program are to:

1) promote collective learning and sharing of best practices in diabetes based on regionally relevant evidence.
2) increase regional awareness about the magnitude of diabetes and its preventable nature through education, ongoing data collection and implementation of evidence-based care.
3) establish a regional diabetes registry
    a) to improve our understanding of disease progression including identification of novel risk factors for diabetes and associated complications.
    b) to monitor levels of adherence to recommended procedures and care processes by people with diabetes and care providers and their changes over time.
    c) to document the patterns of care and levels of attainment of recommended treatment targets and their changes over time.

d) to evaluate the impacts of interventions including use of medications and care protocols on clinical outcomes and their relative cost-effectiveness.

e) to foster inter-sectoral, inter-disciplinary and inter-institutional collaborations to achieve these inter-linked objectives.

4) collect the evidence and inform relevant stakeholders including general public, people with diabetes, healthcare professionals, policy makers and payers, to change policies and practices in order to make quality diabetes care accessible, sustainable and affordable.

Using *state of the art* information technology, the JADE electronic portal provides a virtual platform to enable care professionals to record, manage and analyze the large amount of information collected during various consultation visits. The JADE Program also incorporates risk equations developed and validated by the CUHK Diabetes Care & Research Group to help doctors assess their patients' future event rates (37). This information, displayed in charts and trend lines, aims to motivate behavioral changes and promote dialogues between patients and care providers to set and attain mutually agreeable treatment goals (35).

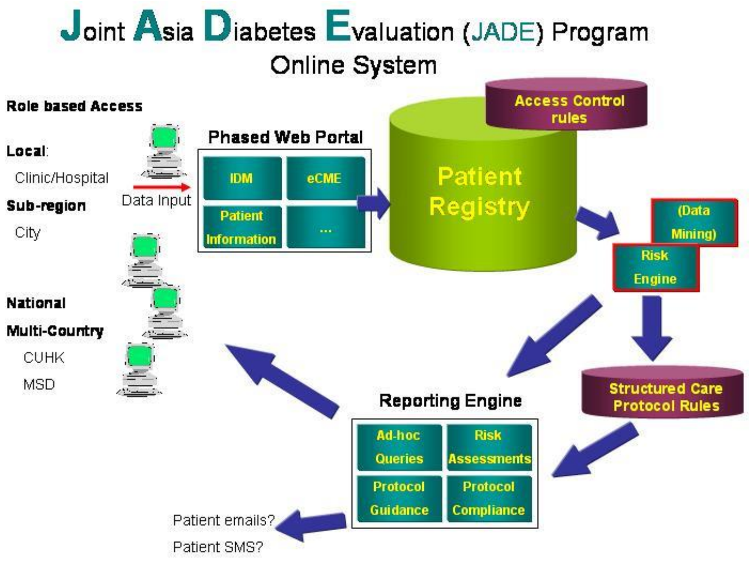

Apart from providing templates for documentation of risk factors and complications using standardized protocols, the JADE Program also incorporates different evidence-based care protocols with recommendations on FU schedules and care processes in accordance to the patient's risk profile. Decision support in terms of prompts, charts, trend lines and practice tips are used to help doctors and patients make informed decisions and optimize care. The JADE Program also possesses matrixes which enable care providers to track clinical progress

and risk factor control for benchmarking and quality improvement purposes. On the other hand, the DIAMOND Program represents the first step towards a quality assurance program by enabling the doctor to collect data during CA at regular intervals to establish a diabetes registry. The ongoing data collection provides an invaluable platform for collaborative epidemiological and interventional studies pertinent to Asian populations (35).

HYPOTHESIS

Delivery of quality diabetes care requires a team approach with informed decisions from patients and care providers. Several lines of evidence suggest that a protocol-augmented collaborative care model with particular focus on periodic assessments, reinforcement of patient compliance and attainment of multiple treatment targets reduces risk of cardiovascular -renal complications and premature death in type 2 diabetes.

We hypothesize that the use of *state of the art* information technology to record, manage and analyze clinical information collected during consultation visits improve the effectiveness and efficiency in implementing these care protocols through decision support and regular feedback to both patients and care team.

We further hypothesize that structured care using the JADE Program delivered by an interdisciplinary team will be more effective than usual care (DIAMOND) Program in controlling multiple risk factors, reducing clinical event rates and improving quality of life.

OBJECTIVES

In this 2-year multicentre, randomized integrated disease management program, we shall use a web-based disease management program to guide comprehensive assessment with or without risk stratification and clinical protocols and to compare the effectiveness of the JADE (structured care) versus DIAMOND Programs (usual care) in

1) reducing all diabetes-related clinical endpoints
2) control of multiple risk factors
3) improving quality of life, psychological well-being and health behaviors.

STUDY DESIGN

This is a multicentre, randomized integrated disease management program to compare the effects of structured care using the JADE portal versus usual care using the DIAMOND portal on incidence of all diabetes-related clinical endpoints in a naturalistic setting.

Ethical approval will be obtained from the local institutional board before commencement of the study. The doctor will explain to eligible patients the rationale for undergoing regular CA to detect silent risk factors and complications and the need to assess the cost-effectiveness of using a team to deliver structured care and empower self-management. With written informed consent and after randomization, a trained nurse will perform the CA using structured protocols in all patients at baseline, 12 and 24 months, further assisted by the doctor and HCA. All patients will be asked to complete simple questionnaires to assess psychological health, quality of life and behaviour during CA visit. The trained nurse will perform simple procedures (e.g. measurement of anthropometric indexes, visual acuity, blood pressure (BP), blood drawing and collecting urine samples).

Apart from assisting the nurse, the HCA will be responsible for entering data, managing the database and maintaining an appointment and filing system. The doctor will focus on taking medical history to diagnose, identify needs and monitor clinical progress. The doctor will also perform eye and feet, and any other relevant, examinations or refer patients to appropriate personnel to undergo these assessments during the CA. All data will be first captured using case report form (CRF) during CA and FU visits followed by data entry. Patients randomized to the JADE group will be followed up 2-4 monthly by a doctor-nurse-HCA team using predefined protocol in group review clinics in order to facilitate group education and promote peer interactions. We expect a doctor-nurse-HCA team to screen 4-6 patients daily and recruit 600 patients within 6-8 months from each centre, giving a total FU period of 2.5-3 years.

SETTING AND PATIENTS

In this program, 600 consecutive type 2 diabetic patients will be recruited from each participating centre during a 6-8 month period, with randomization to either the JADE or DIAMOND group in a 1:1 ratio.

Inclusion criteria

1) Type 2 diabetic patients who are willing or can be persuaded to return for ‘regular’ follow-up at 3-4 monthly intervals
2) Aged ≥18 years
3) Patients with newly diagnosed or established disease, treated with lifestyle modification or blood glucose lowering drugs including oral agents with or without insulin
4) For newly diagnosed type 2 diabetic patients, their plasma glucose levels should be:
    a) Fasting plasma glucose (PG) ≥7.0 mmol/L on 2 or more occasions, and/or
    b) Random or 2-hour PG ≥11.1 mmol/L (after 75 gram oral glucose tolerance test) on 2 or more occasions, and/or
    c) HbA_{1c} ≥6.5%

Exclusion criteria

1) Type 1 diabetes defined as a history of ketosis at diagnosis [acute symptoms with heavy ketonuria (>3+) or ketoacidosis] or continuous requirement of insulin within one year of diagnosis (38).
2) Patients with reduced life expectancy (e.g. less than 6 months) due to recent diagnosis of advanced cancers (e.g. within last 2 years) and other life-threatening conditions.
3) Patients with a mental condition rendering them unable to understand the nature, scope, and possible consequences of the study.
4) Patients actively enrolled in another intervention study.
5) Patients who are unwilling to return for regular follow up.

OUTCOME MEASURES

Composite primary endpoint (CRF (1-5))

1) Incidence of all diabetes-related endpoints including:
    a) cardiovascular events (acute myocardial infarction, revascularisation procedures, heart failure, unstable angina, arrhythmia, stroke, transient ischemic attacks requiring hospital admissions) and related death
    b) chronic kidney disease (eGFR<60 ml/min/1.73m^2) or end stage renal disease (dialysis and/or eGFR<15 ml/min/1.73m^2) and related death
    c) visual impairment (corrected visual acuity of 20/200 or worse) or eye surgery (cataract removal, retinal surgery and vitrectomy)

d) lower extremity amputation or foot ulcers requiring hospitalizations

e) major infections – pulmonary and non-pulmonary requiring hospitalizations

f) all-site cancers

g) death

Composite secondary endpoint

2) Proportions of patients with improved control of risk factors defined as:
   a) 2 or more of the ‘ABC’ targets:
      i) HbA_{1c}<7%
      ii) BP<130/80 mmHg
      iii) LDL-C <2.6 mmol/L
   b) and/or
   c) 2 of the following changes in risk factor control:
      i) at least 0.5% reduction in HbA_{1c}
      ii) at least 5 mmHg reduction in systolic BP
      iii) at least 0.5 mmol/L reduction in LDL-C
      iv) at least 3% reduction in body weight

Composite tertiary endpoint

3) Quality of life (ED-5Q)
4) Frequency of hypoglycaemia (in the last 3 months) as recorded in the JADE CA form
5) Behavioral changes in last 3 months as recorded in the JADE CA form
   a) Frequency of Self Blood Glucose Monitoring
   b) Adherence to balanced eating
   c) Adherence to regular exercise
6) CQ4 for compliance
7) PHQ-9 for depression
8) Hospitalization days and cost-effectiveness analysis
9) Additional questionnaires are available for interested investigators to assess other psychological parameters (self efficacy, diabetes related stress, perception of happiness, personal habits) and self-care activities to explore reasons which may explain differences in outcomes between the 2 groups (see appendix).

STUDY PROTOCOL

Infrastructure, logistics and manpower

The doctor will identify a dedicated space, however small, which can accommodate a couch and office equipment to enable the nurse and HCA to perform clinical assessments, interview patients, and manage a database. The doctor will provide simple equipment and make necessary arrangements to help the nurse complete the CA including eye and feet examination, blood and urine tests. The doctor will emphasize to patient the important role of a nurse in assisting doctors to provide continuous and quality care. Patients enrolled in the program will be given easy access to attend these assessment and education sessions (e.g. pre-booked appointments) to reduce waiting time and default rate.

Randomization

Computer-generated codes numbered 1 to 600 with assignment to either JADE or DIAMOND group will be prepared by ADF for each site. Three sets of envelopes with equal assignment to JADE or DIAMOND Group will be given to the study site at regular time intervals depending on rate of recruitment. The randomization procedures are as follows:

1) The doctor or nurse will explain to eligible patients regarding the international recommendation on performing regular CA to detect silent risk factors and complications.
2) They will also explain to patients the need to assess cost-effectiveness of using a team to implement structured care by comparing the latter with usual care.
3) After the patient has signed the consent, a staff not participating in the study will open the envelope containing the randomization group and sign and date the envelope.
4) The signed envelope will be kept by the nurse in a secure place for audit purpose.
5) The nurse will explain to patient whether they have been randomized to JADE or DIAMOND and the assessment will proceed accordingly.
6) The nurse will fax the randomization form to the ADF office and keep a log of randomized patients and record status including study completion, premature discontinuation, consent withdrawal or lost to follow up.
7) The doctor or nurse will contact the ADF to obtain the user code to enrol the patient in either the JADE or DIAMOND portal and obtain a patient identification number (ID).

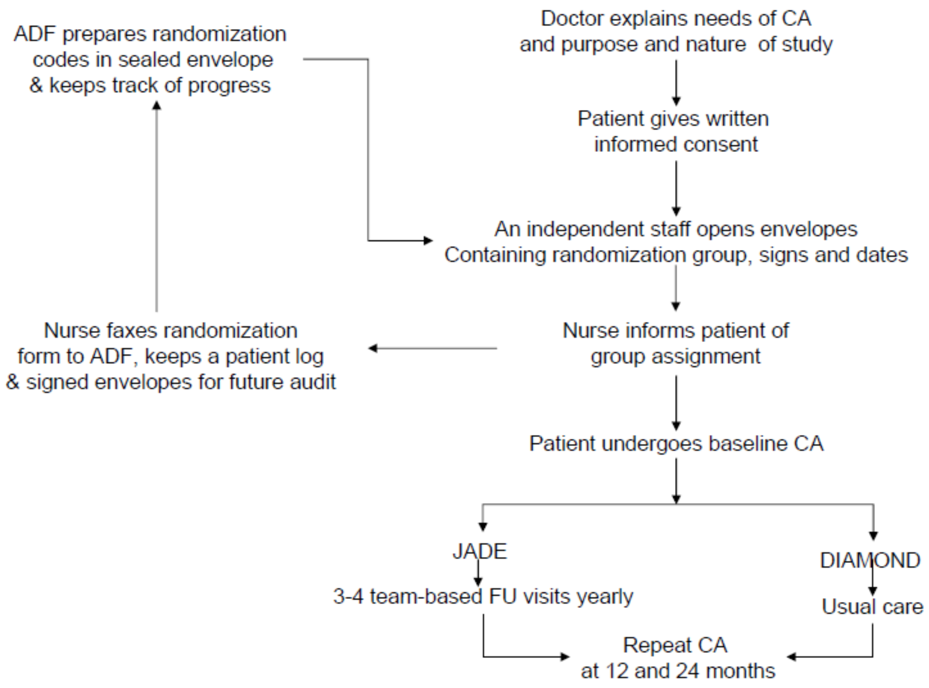

Enrolment

1) Patients newly diagnosed to have or with known history of type 2 diabetes attending the clinic who fulfil the inclusion and have no exclusion criteria will be invited to participate and explained the nature of the AP-JD Program.
2) Patients will be asked to provide written informed consent and give permission to the care team to ascertain clinical status by telephone or email as well as obtain their clinical and biochemical data for analysis and research purpose at least on a yearly basis for 3 years.

Comprehensive assessment

1) The following information will be collected from all patients:
   a) Personal history on occupation, education, smoking and alcohol drinking.
   b) Family history on diabetes and other illnesses.
   c) History of diabetes-related complications, illnesses and symptoms.
   d) Information on diabetes education and self-care.
   e) History of cancers and other medical conditions.
   f) Major clinical events and hospitalizations
   g) Medications
2) Blood pressure and anthropometric measurements including body weight and height, waist and hip circumferences.
3) All patients will attend the CA session after at least 8 hours of fasting for measurement of plasma glucose (PG) and lipids (total cholesterol, triglyceride, HDL-C and LDL-C);

HbA$_{1c}$; renal/liver function test; estimated glomerular filtration rate (eGFR) and complete blood count. A random spot urine will be collected for measurement of albumin-creatinine ratio (ACR). If fasting is not possible, TC and HDL-C are acceptable for lipid profile.

4) The doctor will perform eye (ophthalmoscopy through dilated pupils) and feet examination (reflexes, foot pulses and sensation) or arrange these to be performed by appropriate care professionals.
5) Alternatively, the nurse can be taught to use doppler scan, monofilament and graduated tuning fork to examine the feet.
6) He/she can also be taught how to use fundus camera (if available) to take retinal pictures which will be reviewed by the doctor.
7) The patient will complete the following questionnaires to assess psychological well being and compliance:
   a) Quality of life using ED-5Q (patients' mobility, self-care, usual activities, pain/discomfort and anxiety/depression)
   b) 4-item compliance questionnaire (CQ4)
   c) Depression questionnaire (PHQ9)
8) Depending on the interests of the investigators, there are additional questionnaires which can be used to assess self efficacy, diabetes related distress, personal habits, psychological mood and health perception which can help the care team to provide education and empowerment (see appendix).
9) All patients will undergo repeat CA and complete these questionnaires at 12 and 24 months.
10) Due to the busy nature of the clinic, the nurse and doctor will first enter data using the CA and FU CRFs followed by data entry into the portal.
11) At baseline, year 1 and 2 as well as close out of study, all outcome measures and medications will be ascertained by phone or face to face interview (CRF: 1-5).

Intervention – JADE Group

In the JADE group, the patients are managed by a team of doctor, nurse and HCA, who share different tasks ranging from clinical diagnosis, education, data management and treatment to targets in order to improve the logistics in delivering this complex care protocol, augmented by information technology.

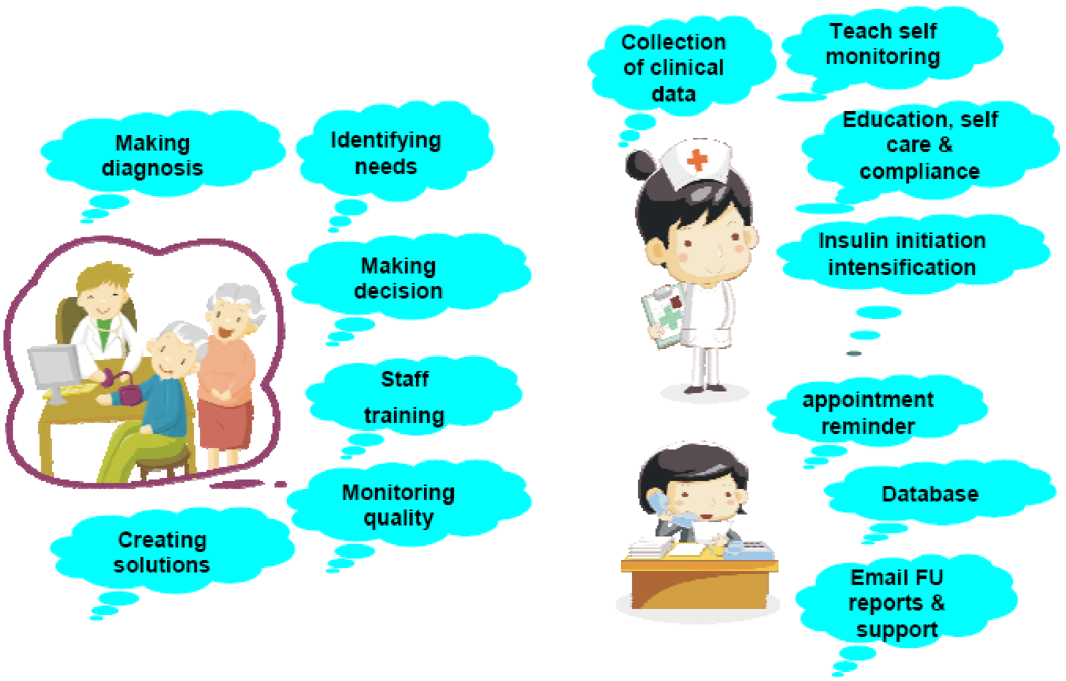

1) Patients randomized to the JADE group will be followed according to the protocol based on their risk levels. In addition to the CA visit, all subsequent FU visits will be documented followed by data entry into the JADE portal with issue of reports to both patients and doctor to promote sharing of information and informed decisions.

Risk stratification	Very-high risk<br>4	High risk<br>3	Medium risk<br>2	Low risk<br>1
Cardiovascular renal complication	≥ 1	0	0	0
Any risk score	NA	≥ SP	≥ ST and < SP	< ST
Stratification parameters	NA	≥ 3	2	0–1
eGFR	NA	< 60	60–< 90	≥ 90
Recommended number of medical visits per year	6 or more	4 or more	3 or more	At least 2
Comprehensive assessment	Every 12–18 months			
Other recommendations	Counselling/laboratory tests/telephone calls between medical visits			

Parameters for risk stratification:
1 Cardiovascular-renal complications: cardiovascular diseases (stroke, coronary heart disease and/or peripheral vascular disease with or without interventions or medications), heart failure and end-stage renal diseases [on renal replacement therapy or estimated glomerular filtration rate (eGFR) < 15 ml/min 1.73/m^2).
2 Risk score: high sensitivity (ST) and specificity (SP) cut-off points, derived from each of the four risk equations of the Hong Kong Diabetes Registry, were used to estimate probability of risk of clinical outcomes.
3 Stratification parameters: Current or ex-smoker; body mass index ≥ 27.5 kg/m^2 or waist circumference ≥ 80 cm in women or ≥ 90 cm in men; blood pressure > 130/80 mmHg or treatment with antihypertensive drugs; low-density lipoprotein-cholesterol (LDL-C) > 2.5 mmol/l; triglyceride (TG) ≥ 2.3 mmol/l and/or high-density lipoprotein-cholesterol (HDL-C) < 1 mmol/l; treatment with lipid-regulating drugs if LDL-C ≤ 2.5 mmol/l and TG < 2.3 mmol/l and HDL-C ≥ 1.0 mmol/l; random spot urinary albumin–creatinine ratio > 3.5 mg/mmol in women or > 2.5 mg/mmol in men; foot at risk defined by two of three of the following: sensory neuropathy, skin changes (e.g. fungal infection, dry skin) or deformities (e.g. claw feet or hallux deformities); retinopathy; HbA_{1c} ≥ 8%.
4 eGFR in ml/min 1.73/m^2.
Risk levels:
1 Level 4: Presence of any cardiovascular or renal complication.
2 Level 3: No cardiovascular or renal complication; and either having three or more stratification parameters, or risk scores above the high specificity cut-off in any one of the risk equations, or eGFR < 60 ml/min 1.73/m^2.
3 Level 2: No cardiovascular or renal complication; none of the conditions (risk score, stratification parameters, eGFR) defined in the 'High risk' category but not belonging to the 'Low risk' category.
4 Level 1: No cardiovascular or renal complication; and having other condition fulfilling the 'Low risk' (one or less stratification parameter, and risk scores below the high sensitivity cut-off in all of the risk equations, and eGFR ≥ 90 ml/min 1.73/m^2).

2) After the patient has undergone CA, the nurse will arrange 2-4 hours of diabetes education in groups or individually and explain the various features of the JADE report.

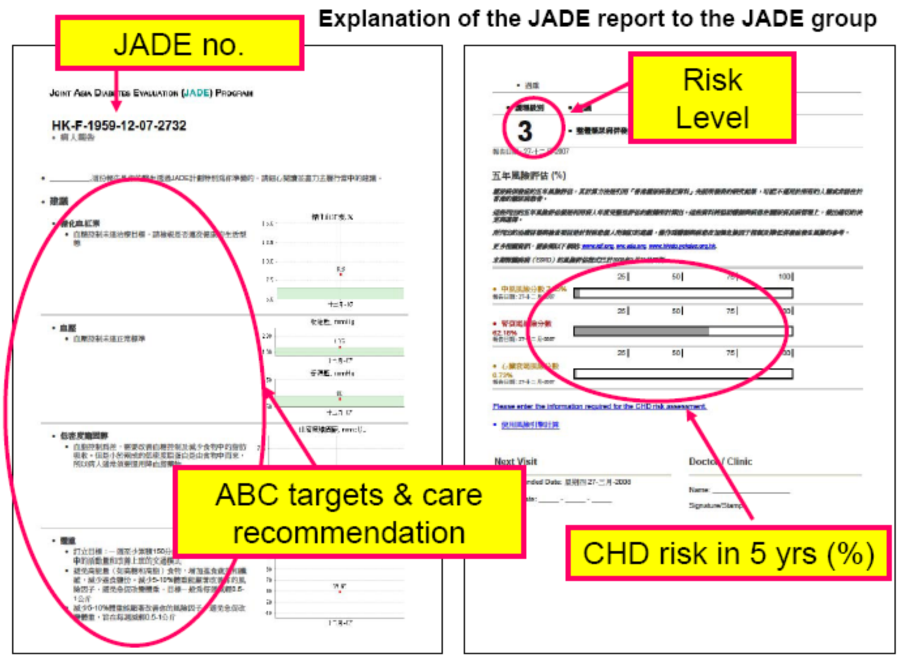

3) If not yet put in place, the nurse/HCA will prepare a clinic folder for each patient and create a system to facilitate appointment booking and tracking of default.
4) Patients are booked for reviews led by the doctor-nurse-HCA team every 2-4 months, preferably in a setting different from the busy clinics in order to facilitate group education and promote peer support.
5) Between each FU visit, the nurse or HCA will contact the patient by phone or email to remind them of the appointments (e.g. medical FU visit or laboratory tests), adhere to medications and healthy lifestyles, perform self glucose monitoring and provide psychosocial support, as appropriate.
6) Before each FU visit, the nurse will ensure all tests (e.g. HbA_{1c}, lipids, blood haemoglobin, renal function etc) ordered by the doctor are available in the folder for decision making.
7) At each FU visit, the patients will first see the nurse for record of BP, body weight and blood glucose (or HbA_{1c}) as appropriate and enter them in the CRF. Patient’s compliance will also be checked at each visit.
8) After the FU visit, the patients will see the nurse to clarify any issues and concerns. The nurse will reinforce compliance and record any changes in medications and note any procedures or tests ordered by the doctor. As and when appropriate, the nurse will remind doctors on action items prompted by the JADE e-portal, if omitted.
9) After each FU visit, the nurse will issue summary reports to be given to patients and doctors to promote sharing of information.

10) The FU and CA reports can be given to the patient on the same day of the visit or emailed or sent by post to patient as appropriate.

Intervention - DIAMOND group

Patients randomized to the DIAMOND group will receive usual care after the initial CA with repeat CA at 12 and 24 months.

Manpower estimation

From each participating centre, 600 consecutive type 2 diabetic patients will be recruited. Each day, approximately 4-6 patients can undergo CA performed by the doctor-nurse-HCA team. We expect to complete recruitment in 6-8 months with 300 patients randomized to the usual care (DIAMOND) and 300, to structured care (JADE) (see example for suggestion of workflow).

	**Mon**	**Tues**	**Wed**	**Thurs**	**Fri**
**AM**	CA<br>of 4-6 patients	CA<br>of 4-6 patients	CA<br>of 4-6 patients	CA<br>of 4-6 patients	CA<br>of 4-6 patients
**PM**	Data entry<br>Reminder<br>Education	FU clinic of 10<br>JADE patients by<br>trio-team	Data entry<br>Reminder<br>Education	FU clinic of 10<br>JADE patients by<br>trio-team	Data entry<br>Reminder<br>Education

Assuming 4 patients daily and 20 patients weekly, 600 patients will be enrolled in 30 weeks. On average, each patient has 4 team-based visits yearly to give 1200 patient-visits. Assuming 10 patients per session, this will give 120 clinic sessions, i.e. 2 clinics weekly. The nurse-HCA will use other sessions to educate patients, manage data, recall default, check protocol adherence and risk factor control, as guided by the doctor.

Logistics and timeline

The following diagram summarises the flow of the study including approval of local institutional board, staff recruitment and training, use of portal and patient enrolment. We expect completion of recruitment within 6-8 months after all logistics are put in place. All patients will be followed up for at least 24 months with completion of follow up of all randomized patients, 3 years after the first patient is entered the study.

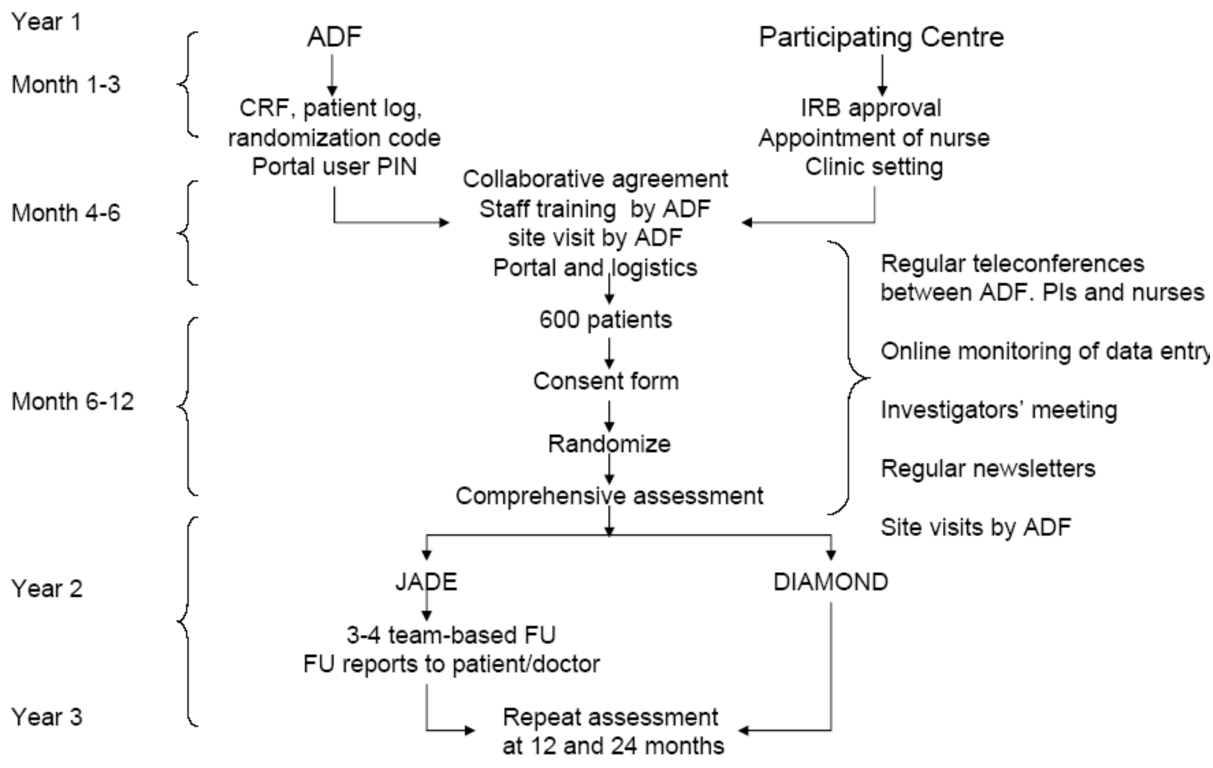

SAMPLE SIZE CALCULATION

In this multicentre study enrolling 600 patients from sites with different health settings in Asia Pacific region, we aim to test the hypothesis whether using a doctor-nurse-HCA team will reduce all diabetes-related clinical endpoints and improve control of risk factors compared to usual care, in low, middle and high-income areas including public and private clinics as well as hospital or community-based clinics.

Primary composite outcome

The following table summarises the hazard ratios (HR) of different diabetes-related endpoints from 3 studies (16; 20; 29), all of which have used a team approach to manage medium to high risk type 2 diabetic patients from Asia and Europe, using predefined protocols and targets. These results confirm the inter-ethnic differences in disease predisposition with Caucasians being more likely to suffer from cardiovascular disease and Chinese, from renal disease (39). Despite these differences, in all 3 studies, the HR ranged from 0.3 to 0.7 for all diabetes-related events in the team-based structured care group compared to usual care.

Summary of RCT data and 3 studies shoing the effects of risk factor reduction and team-based care on clinical outcomes

Steno study		*baseline*	*7 years*	*13 years*	*Difference at 7 yr*
LDL-C	SC	3.44	2.15	1.84	
	UC	3.54	3.3	1.99	1.1 (30%)
TG	SC	1.8	1.3	1.12	
	UC	2.3	1.8	1.67	0.5 (30%)
A1c	SC	8.4	7.9	7.7	
	UC	8.8	9	8	1 (12%)
SBP	SC	146	131	140	
	UC	149	146	146	15 (10%)
DBP	SC	85	73	74	
	UC	86	78	73	5 (5%)
ACEI	SC	20	97	91	
	UC	19	70	87	
Statin	SC	0	85	84	
	UC	3	22	82	

RCT data	*Risk factor*	*changes*	*risk reduction*	*Remarks*
	LDL-C	30%	20-30%	CVD
	SBP	14 mm Hg	12%	CVD
	A1c	1%	12-14%	CVD
			21%	all DM endpoint
			21%	all DM death
			19%	cataract
			37%	microvascular
			43%	PVD

Steno 13 year FU	*SC=80*		*UC=80*		*HR*
death	24	0.30	40	0.50	0.60
CVD-death	9	0.11	18	0.23	0.50
MI	8	0.10	21	0.26	0.38
stroke	6	0.08	18	0.23	0.33
PCI	8	0.10	13	0.16	0.62
revascularization	6	0.08	10	0.13	0.43
amputation	6	0.08	14	0.18	0.43
all CVD events	25	0.31	48	0.60	0.52
cancer-death	2	0.03	5	0.06	0.40
DM nephropathy	20	0.22	37	0.46	0.48
ESRD	1	0.01	6	0.08	0.17
Blindness	2	0.03	7	0.09	0.29
Progression of DMR	41	0.51	54	0.68	0.76
Laser or macular edema	14	0.18	27	0.34	0.52

2-year SURE Study	*>=3 target*		*<3 targets*		*HR*
	91		114		
ESRD/death/dSeCr	14	0.15	34	0.30	0.52
dialysis	6	0.07	12	0.11	0.63
dSeCr	10	0.11	21	0.18	0.60
death	4	0.04	15	0.13	0.33
MI/angina/PCI	4	0.04	7	0.06	0.72
heart failure	12	0.13	16	0.14	0.94

7-year AJMC paper	*SC*		*UC*		*HR*
	91		80		
death	8	0.09	20	0.25	0.35
death/CVS/renal	20	0.22	30	0.38	0.59

These Kaplan-Miere plots show the estimated events from these 3 studies in a 2-year period.

Chinese T2D patients with CKD (eGFR 30 ml/min/1.73m^2)
Chan JC et al Diabetes Care 2009

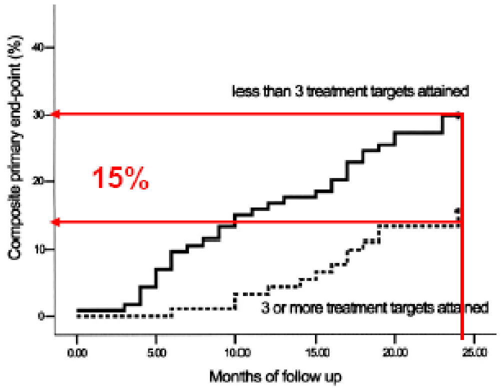

Chinese T2D patients with HT (no CVD/CKD)
So WY et al Am J Managed Care 2003

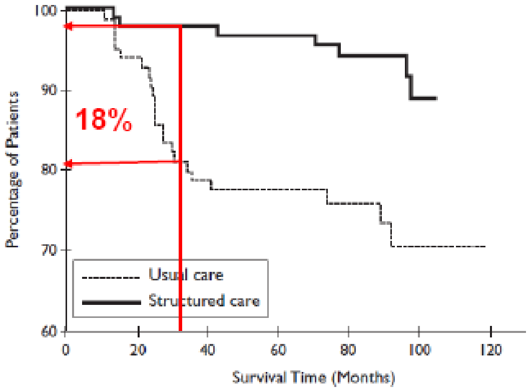

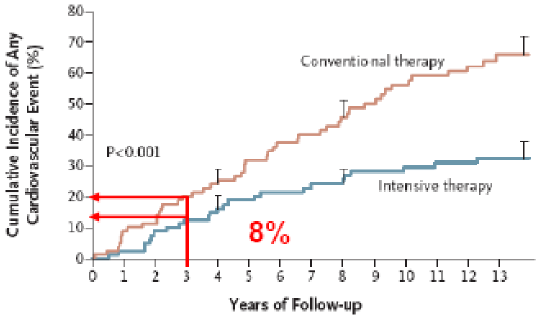

Caucasian T2D patients with microalbuminuria (no CVD/CKD)
Gaede P et al NEJM 2008

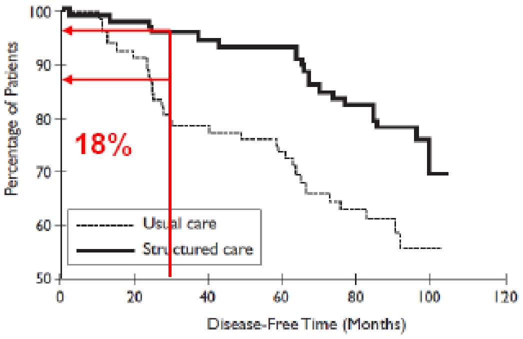

Using validated risk equations and risk parameters, the JADE Program stratified patients into very high risk, high risk, medium risk and low risk groups with respective estimated annual event rate of 7%, 3%, 1.5% and 1%.

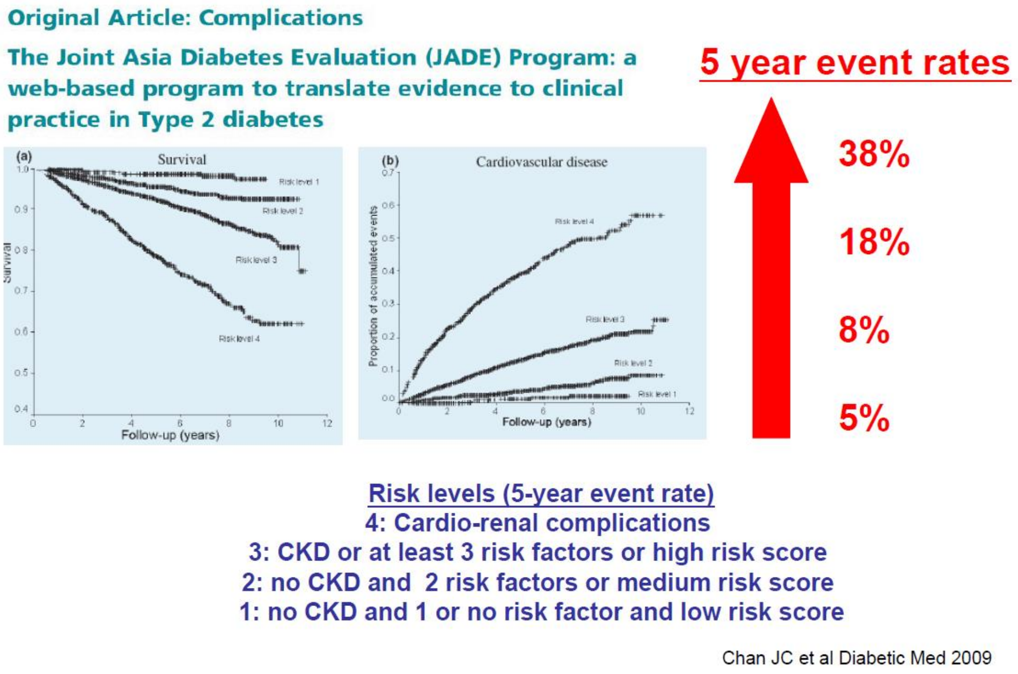

In our first year analysis of the JADE Asia Pacific Program, the majority of enrolled patients from 7 countries belonged to very high to high risk category.

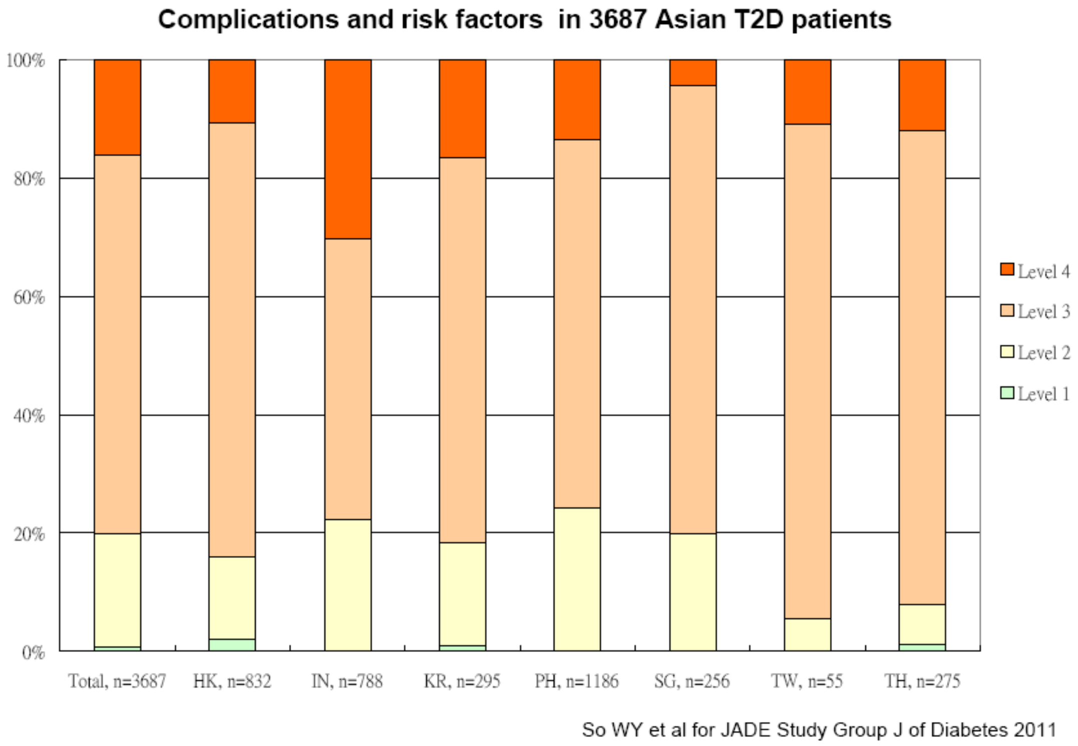

We used these observations to estimate the sample size needed to give a 2-year study 80% power with an alpha value of 0.05 to confirm the superiority of team-based structured care over usual care on clinical endpoints. We used different combinations of scenarios including an annual hazard rate ranging from 0.01 to 0.10 in the usual care group and a HR ranging from 0.5 to 0.85 in the structured care group with an attrition rate of 0.2, to estimate the sample size. Using the most conservative scenario where the annual hazard rate is 0.02, i.e. 0.04 in 2 years and assuming a HR of 0.8 in the structured care group, the study will have sufficient power to confirm the primary hypothesis. For high risk patients with a hazard rate of 0.05 and those with medium risk (0.03), and assuming a modest HR of 0.8 in the structured care group, a sample size of 6626-13762 patients will give the study 80% power at an alpha value of 0.05.

80% power at 0.05 alpha						
minimum 2 year follow up						
LOW RISK				VERY LOW RISK		
hazard ratio	0.5	0.8	0.85	0.5	0.8	0.85
annaul hazard rate	0.02	0.02	0.02	0.01	0.01	0.01
hazard rate (usual)	0.04	0.04	0.04	0.02	0.02	0.02
hazard rate (structured)	0.02	0.032	0.034	0.01	0.016	0.017
absolute difference	0.02	0.008	0.006	0.01	0.004	0.003
sanple size in each group	1239	8760	15868	2515	17838	32333
total sample size	2478	17520	31736	5030	35676	64666
assuming 20% attrition rate	2974	21024	38083	6036	42811	77599
number needed to treat	50	125	167	100	250	333
MODERATE RISK						
hazard ratio	0.5	0.8	0.85			
annaul hazard rate	0.03	0.03	0.03			
hazard rate (usual)	0.06	0.06	0.06			
hazard rate (structured)	0.03	0.048	0.051			
absolute difference	0.03	0.012	0.009			
sanple size in each group	1067	5734	13820			
total sample size	2134	11468	27640			
assuming 20% attrition rate	2561	13762	33168			
number needed to treat	33	83	111			
HIGH RISK				VERY HIGH RISK		
hazard ratio	0.5	0.8	0.85	0.5	0.8	0.85
annaul hazard rate	0.05	0.05	0.05	0.1	0.1	0.1
hazard rate (usual)	0.1	0.1	0.1	0.2	0.2	0.2
hazard rate (structured)	0.05	0.08	0.085	0.1	0.16	0.17
absolute difference	0.05	0.02	0.015	0.1	0.04	0.03
sanple size in each group	474	3313	5988	219	1497	2695
total sample size	948	6626	11976	438	2994	5390
assuming 20% attrition rate	1138	7951	14371	526	3593	6468
number needed to treat	20	50	67	10	25	33

Secondary composite outcome

In a 1-year pilot study, 240 high risk Chinese T2D patients with CHD or multiple risk factors who have undergone CA using the JADE portal were randomized to usual care or structured

care group. In the latter group, supported by an endocrinologist and guided by a protocol, a diabetes nurse, assisted by a HCA, reviewed these patients every 3 to 4 months in addition to receiving usual care. At the end of 1 year, there were significant reductions in all risk factors in both groups, but more so in the team-based structured care group. The rate of attaining at least 2 of the 3 'ABC targets' increased by 2.5 fold from 15% to 37% in the structured care group while respective figures in the usual care were 20% to 28% with 1.4 fold increase, giving a 2 fold difference between the 2 groups. In the Hong Kong Diabetes Registry, type 2 diabetic patients who attained 2 of the 3 'ABC' targets had 50% risk reduction in incident CHD after a mean follow up period of 6 years (40).

**Change in risk factors at 1 year**	**HbA_{1c} (%)**		**Systolic BP (mmHg)**		**LDL-C (mmol/l)**		**body weight (%)**	
Effect size (reduction compared to baseline)	0.5	1	5	10	0.5	1	3	5
Structured care (%)	77	61	46	35	32	16	16	8.7
Usual care (%)	60	44	29	18	26	9	6	2.6
Between group Difference (%)	17	17	17	17	6	7	10	6

In a multicentre randomized study involving Chinese type 2 diabetic patients with chronic kidney disease, 61% of patients receiving team-based structured care achieved 3 or more treatment goals (HbA_{1c}<7%, LDL-C<2.6 mmol/l, BP<130/80 mmHg, triglyceride<2 mmol/l and persistence with ACE inhibitor or AII receptor blockers) compared to 28% in the usual care group, i.e. 2-fold increase. Patients who attained 3 or more treatment targets had 50% risk reduction of dialysis or ESRD at 2 year (29).

Based on these observations, we assume that the rate of attaining at least 2 'ABC' goals will increase by 2-fold compared to 50% in the usual care group and assuming 20% attrition rate, we estimate that 295-1814 patients will give the study sufficient power depending on the baseline rate of attaining 2ABC goals. In the first year analysis of the Asia Pacific JADE Program, 12-36% of patients attained at least 2 treatment goals with an average value of 30%.

	Total (3687)	HK (832)	India (788)	Korea (295)	Ph (1186)	Sing (256)	TW (55)	Thai (275)
HbA_{1c}<7%	35.3	61.8	13.8	40.7	31.3	35.2	25.5	29.8
BP<130/80 mmHg	32.3	45.6	26.0	38.3	27.2	12.9	34.6	42.9
LDL-C<2.6 mmol/L	34.0	39.2	44.8	33.6	20.5	29.7	20.0	52.7
No target achieved	32.6	14.3	38.3	28.1	42.2	42.8	40.0	23.3
Any 1 target achieved	38.7	37.1	40.6	36.6	39.2	37.9	43.6	37.5
Any 2 targets achieved	23.4	36.3	19.2	29.8	16.2	15.2	12.7	29.8
All 3 targets achieved	5.4	12.3	1.9	5.4	2.5	3.1	3.6	9.5

We also estimate the sample size needed to confirm the benefits of team-based care on controlling multiple risk factors. Apart from LDL-C goal, which can readily be achieved by initiating patients on statin therapy and thus requires a larger number to detect the small difference between the usual care and structured care groups, we estimate that 300-600 patients will be needed to confirm the superior effects of structured care. Given the expected heterogeneity in clinic settings, patient profiles and health care systems amongst different sites, a sample size of 600 patients will give each site sufficient power to confirm the superior effects of team-based structured care within their own clinic setting.

% of patients at 2 ABC goals in JADE-AP	0.36	0.3	0.12
SC (increased by 2-fold)	0.72	0.6	0.24
UC (increased by 50%)	0.54	0.45	0.18
absolute difference	0.18	0.15	0.06
80% at 0.05 alpha each group	123	186	756
total sample size	246	372	1512
assuming 20% attrition rate	295	446	1814
number needed to treat	6	7	17

Risk factors	A1c	A1c	SBP	SBP	LDL-C	LDL-C	BW	BW
units	%	%	mmHg	mmHg	mmol/l	mmol/l		
reduction in risk factors compared to baseline	0.5	1	5	10	0.5	1	0.03	0.05
proportion of patients in SC	0.77	0.61	0.46	0.35	0.32	0.16	0.16	0.087
proportion of patients in UC	0.6	0.44	0.29	0.18	0.26	0.09	0.06	0.026
absolute difference	0.17	0.17	0.17	0.17	0.06	0.07	0.1	0.061
80% at 0.05 alpha each group	128	146	138	117	930	378	172	256
total sample size	256	292	276	234	1860	756	344	512
assuming 20% attrition rate	307	350	331	281	2232	907	413	614
number needed to treat	6	6	6	6	17	14	10	16

Validation of risk equations derived from the Hong Kong Diabetes Registry

Assuming a 2% annual incidence of all diabetes-related event rates including death, stroke, heart disease, lower extremity amputation, visual impairment, renal failure and cancer, i.e. 4% in 2 years, a sample size of 596 will confirm this estimate with an error of 1% for each

participating centre. In our previous analysis, we have shown that the UKPDS risk engine under-estimated the stroke rate and, over-estimated the CHD rates in Chinese type 2 diabetic patients. Furthermore, compared to our risk equations, UKPDS does not utilize important risk parameters such as eGFR, ACR and blood hemoglobin which are powerful predictors of clinical endpoints in Asian populations (41) (42).

DATA ANALYSIS

All data will be analyzed using the SPSS statistical software (Windows 13.0 version, Chicago, USA). A p value of 0.05 or less (2-tailed) will be considered significant. For the primary composite endpoint, an intention to treat analysis will be performed including all patients randomized to both groups who have at least returned for one visit or contacted by phone once post randomization to ascertain clinical status (alive, dead or hospitalized). Descriptive analysis will be used to report all clinical and laboratory parameters. Student's t-test, Chi-squared test and Analysis of Co-variance will be used for between-group and within-group comparisons for all baseline data and endpoints, as appropriate. Kaplan-Meier curve analysis will be used to compare the incidence of primary composite endpoint between the JADE and DIAMOND group adjusted for age, sex, ethnicity and disease duration. Interaction between group assignment and covariates including age, gender, duration of diabetes (< or ≥8 years), socioeconomic group, complications, risk factors, JADE risk categories, compliance and psychological health parameters will be examined. Logistic regression will be used to determine independent predictors for achieving primary composite endpoint using all relevant covariates including age, gender, duration of diabetes (< or ≥8 years), centre characteristics, socioeconomic group, risk levels, metabolic and risk factor control, compliance, self care, psychological health parameters and intervention groups.

A per protocol analysis will be performed in the JADE group with details of intervention, where we shall compare baseline clinical profile, care processes, number of visits and use of medications between patients who develop and those who do not develop clinical endpoints. A sensitivity analysis will also be performed using hazard rates of the JADE and DIAMOND group amongst those who have defaulted.

DEFAULT AND MISSING DATA

This is a translational research which aims to evaluate the effectiveness of team-based, protocol-augmented care on clinical endpoints in a naturalistic environment. No new investigational drugs will be used and all care processes are recommended by international guidelines. Given the diversity of health care systems in these countries, investigators will be asked to first define the processes of ‘usual care’ in their clinics. We shall also assess the needs and feasibility of implementing team-based protocol driven care in their clinic setting (see appendix). To reduce the number of defaulters in the JADE and DIAMOND groups, the doctor and nurse will explain clearly the nature of the program and seek their consent for contacts via phone or email or letter regarding clinical status, notably death or major clinical events or hospitalizations at least once yearly even if they are not able to return for repeat CA.

SITE VISITS AND EVENT ADJUDICATION

An event monitoring and adjudication committee consisting of international experts will be formed to review the narratives and supporting documents for pre-defined clinical events. The ADF project team will conduct training programs to explain rationale, implementation and expected results of the study, followed by periodic visits and teleconferences to ensure data integrity and adherence to good clinical practice. An annual investigator meeting will be held for investigators to share experiences in implementing structured and collaborative care and to promote cooperative learning.

BENEFITS AND SIGNIFICANCE

Despite the increasingly popular use of electronic health record (eHR) systems to improve quality of care in different health institutions, most of these systems only record data rather than utilize them in a systematic manner to inform care providers and patients regarding their health status, targets and progress. The JADE Program is an innovative program which combines a change in clinic practice environment, augmented by information technology, to facilitate decision making with ongoing evaluation. Given the resource implications in developing these complex systems, evaluation of its impacts on clinical outcomes and its cost-effectiveness will provide a useful blueprint for integrating these features into future systems to redesign current health care models, in order to bring out the best of technology

and clinical care.

The implementation of the AP-JD Program will establish a prospective cohort of type 2 diabetic patients (Asia Pacific Diabetes Registry) for documentation of pattern of risk factors, complications, quality of care, psychological well being and drug use in Asia Pacific region. Through this multicentre program, a group of thought leaders and trio-teams will be established in the region with infrastructure and experience to use structured and collaborative care to improve clinical outcomes in diabetic patients. Apart from enabling doctors to establish a disease registry for quality assurance at his/her clinic, the JADE Program will provide a useful tool to benchmark care standards, evaluate cost-effectiveness of drug and non-drug based interventions in real practice and identify unmet needs.

From a more pragmatic perspective, while there is growing consensus that collaborative and protocol-driven care may offer the ultimate solution to reduce the burden of silent chronic diseases such as diabetes, to date, there are no clear guidelines on how this can be implemented in practice in an affordable and sustainable manner. In this innovative translational project, we have shared our experiences in developing this model in Hong Kong during the last 2 decades which has now been adapted by the Hong Kong Government as standard practice and policy. We have further capitalized on our learning since the launch of the JADE Program in 2007 to design this multicentre program with detailed descriptions of manpower estimation, infrastructure, logistics and costs needed to realize structured collaborative care in real practice and in different care settings.

To date, we have enrolled more than 20,000 patients in the JADE Program. Our preliminary analysis suggested that the JADE report has motivated behavioral changes in both patients and doctors resulting in improved care standards. Using our Nurse Consultant Clinic as an example, all 242 patients with heart disease underwent comprehensive assessments using the JADE protocol and all referring doctors were given detailed JADE reports with charts and decision supports. All patients also received a similar JADE report written in Chinese language. At one year, all patients had significant improvements in ABC targets with further improvement in the group managed by a trio team of nurse, HCA and endocrinologist. This is in stark contrast to most audit reports which have shown that, on average, it took 12-18 months before treatments were escalated despite suboptimal control, irrespective whether the

patients were managed by primary care doctors or specialists (44).

Taken together, these preliminary findings strongly suggest that patients, doctors and care professionals may improve knowledge, skills and attitudes by participating in the JADE Program to improve control of risk factors and possibly clinical outcomes. Thus, if these findings can be confirmed in this multicentre program, the JADE Program will change the paradigm in chronic disease management from one of fragmentation to that of integration encompassing early detection, registry enrolment, regular risk assessments, team-based management, regular feedback and decision supports. This mode of care is not dissimilar to the standard practice of having a midwife to look after a pregnant woman and enrolling patients with notifiable diseases such as tuberculosis into a disease registry.

INTELLECTUAL PROPERTY

ADF will provide the JADE or DIAMOND electronic portals to the site without any charge to implement the AP-JD Program. All patients will be managed in accordance to the local standards of practice and financial arrangement including but not limited to consultations, hospitalizations, investigations and medication fees. The combined database will remain the intellectual property of ADF. The ADF project team will perform the analysis and prepare the report under the guidance of the steering committee. While all investigators can eventually use the data from their own clinic for reporting and publication purpose, this will be permitted only after publication of the primary results of the multicentre study and with approval from the steering committee.

ABOUT ASIA DIABETES FOUNDATION

The Asia Diabetes Foundation (ADF) is a non-profit making organization developed to initiate and implement medical, scientific and academic research activities to collect and translate current evidence into prevention and control strategies for diabetes and other chronic diseases.

Our vision is to use pragmatic research and a multidisciplinary approach, augmented by the latest technologies, to promote informed decision making in order to enhance the sustainability, affordability and accessibility of chronic care.

Our mission is to raise awareness, empower patients and those at risk to be proactive in managing their own health needs. Using *state-of-the-art* information technology and evidence-based protocols, we shall support healthcare professionals to use an interdisciplinary approach to act swiftly to detect those at risk and reduce the adverse consequences of diabetes and chronic diseases.

REFERENCES

1. Chan JC, Malik V, Jia W, Kadowaki T, Yajnik CS, Yoon KH, Hu FB: Diabetes in Asia: epidemiology, risk factors, and pathophysiology. *Jama* 301:2129-2140, 2009
2. Roglic G, Unwin N, Bennett P, Mathers C, Tuomilehto J, Nag S, Connolly V, King H: The burden of mortality attributable to diabetes: realistic estimates for the year 2000. *Diabetes Care* 28:2130-2135, 2005
3. Seshasai SR, Kaptoge S, Thompson A, Di Angelantonio E, Gao P, Sarwar N, Whincup PH, Mukamal KJ, Gillum RF, Holme I, Njolstad I, Fletcher A, Nilsson P, Lewington S, Collins R, Gudnason V, Thompson SG, Sattar N, Selvin E, Hu FB, Danesh J: Diabetes mellitus, fasting glucose, and risk of cause-specific death. *N Engl J Med* 364:829-841, 2011
4. Yoon KH, Lee JH, Kim JW, Cho JH, Choi YH, Ko SH, Zimmet P, Son HY: Epidemic obesity and type 2 diabetes in Asia. *Lancet* 368:1681-1688, 2006
5. Wild S, Roglic G, Green A, Sicree R, King H: Global prevalence of diabetes: estimates for the year 2000 and projections for 2030. *Diabetes Care* 27:1047-1053, 2004
6. Yang W, Lu J, Weng J, Jia W, Ji L, Xiao J, Shan Z, Liu J, Tian H, Ji Q, Zhu D, Ge J, Lin L, Chen L, Guo X, Zhao Z, Li Q, Zhou Z, Shan G, He J: Prevalence of diabetes among men and women in China. *N Engl J Med* 362:1090-1101, 2010
7. Yach D, Leeder S, Bell J: Response to chronic versus acute diseases. *Science* 309:380, 2005
8. Narayan KMV, Gregg EW, Engelgau MM, Moore B, Thompson TJ, Williamson DF, Vinicor F: Translation research for chronic diseases. The case for diabetes. *Diabetes Care* 23:1794-1798, 2000
9. Grol R, Grimshaw J: From best evidence to best practice: effective implementation of change in patient's care. *Lancet* 362:1225-1230, 2003
10. Ziemer DC, Doyle JP, Barnes CS, Branch WT, Jr., Cook CB, El-Kebbi IM, Gallina DL, Kolm P, Rhee MK, Phillips LS: An intervention to overcome clinical inertia and improve diabetes mellitus control in a primary care setting: Improving Primary Care of African Americans with Diabetes (IPCAAD) 8. *Arch Intern Med* 166:507-513, 2006
11. Ho PM, Rumsfeld JS, Masoudi FA, McClure DL, Plomondon ME, Steiner JF, Magid DJ: Effect of medication nonadherence on hospitalization and mortality among patients with diabetes mellitus. *Arch Intern Med* 166:1836-1841, 2006
12. Saaddine JB, Cadwell B, Gregg EW, Engelgau MM, Vinicor F, Imperatore G, Narayan KM: Improvements in diabetes processes of care and intermediate outcomes: United States, 1988-2002. *Ann Intern Med* 144:465-474, 2006
13. Ray KK, Seshasai SR, Wijesuriya S, Sivakumaran R, Nethercott S, Preiss D, Erqou S, Sattar N: Effect of intensive control of glucose on cardiovascular outcomes and death in patients with diabetes mellitus: a meta-analysis of randomised controlled trials. *Lancet* 373:1765-1772, 2009
14. Cushman WC, Evans GW, Byington RP, Goff DC, Jr., Grimm RH, Jr., Cutler JA, Simons-Morton DG, Basile JN, Corson MA, Probstfield JL, Katz L, Peterson KA, Friedewald WT, Buse JB, Bigger JT, Gerstein HC, Ismail-Beigi F: Effects of intensive blood-pressure control in type 2 diabetes mellitus. *N Engl J Med* 362:1575-1585, 2011
15. Ting RZ, Yang X, Yu LW, Luk AO, Kong AP, Tong PC, So WY, Chan JC, Ma RC: Lipid control and use of lipid-regulating drugs for prevention of cardiovascular events in Chinese type 2 diabetic patients: a prospective cohort study. *Cardiovasc Diabetol* 9:77, 2011
16. Gaede P, Lund-Andersen H, Parving HH, Pedersen O: Effect of a multifactorial intervention on mortality in type 2 diabetes. *N Engl J Med* 358:580-591, 2008

17. Luk A, Chan JC: Diabetic nephropathy--what are the unmet needs? *Diabetes Res Clin Pract* 82 Suppl 1:S15-20, 2008
18. Kearney PM, Blackwell L, Collins R, Keech A, Simes J, Peto R, Armitage J, Baigent C: Efficacy of cholesterol-lowering therapy in 18,686 people with diabetes in 14 randomised trials of statins: a meta-analysis. *Lancet* 371:117-125, 2008
19. Chan JC: What have we learnt from recent blood glucose lowering megatrials. *Journal Diabetes Investigation*:doi: 10.1111/j.2040-1124.2010.00063.x, 2011
20. So WY, Tong PC, Ko GT, Leung WY, Chow CC, Yeung VT, Chan WB, Critchley JA, Cockram CS, Chan JC: Effects of protocol-driven care versus usual outpatient clinic care on survival rates in patients with type 2 diabetes. *Am J Manag Care* 9:606-615, 2003
21. Leung WY, So WY, Tong PC, Lo MK, Lee KF, Ko GT, Chan WB, Cockram CS, Brenner BM, Shahinfar S, Critchley JA, Chan JC: The renoprotective effects of structured care in a clinical trial setting in type 2 diabetic patients with nephropathy. *Nephrol Dial Transplant* 19:2519-2525, 2004
22. Shojania KG, Ranji SR, McDonald KM, Grimshaw JM, Sundaram V, Rushakoff RJ, Owens DK: Effects of quality improvement strategies for type 2 diabetes on glycemic control: a meta-regression analysis. *JAMA* 296:427-440, 2006
23. Ellis SE, Speroff T, Dittus RS, Brown A, Pichert JW, Elasy TA: Diabetes patient education: a meta-analysis and meta-regression. *Patient Educ Couns* 52:97-105, 2004
24. Li R, Zhang P, Barker LE, Chowdhury FM, Zhang X: Cost-effectiveness of interventions to prevent and control diabetes mellitus: a systematic review. *Diabetes Care* 33:1872-1894, 2011
25. Piwernetz K, Home PD, Snorgaard O, Antsiferov M, Staehr-Johansen K, Krans M: For the DiabCare Monitoring Group of the St. Vincent Declaration Steering Committee. Monitoring the targets of the St. Vincent declaration and the implementation of quality management in diabetes care: the DiabCare initiative. *Diabetic Medicine* 10:371-377, 1993
26. IDF Clinical Guidelines Task Force: Global guideline for type 2 diabetes: recommendations for standard, comprehensive, and minimal care. . *Diabet Med* 23:579-593., 2006
27. So WY, Chan JC: The role of the multidisciplinary team. In *Textbook of Diabetes* Holt R, Cockram S, Flyvbjerg A, Goldstein B, Eds., Blackwell, 2010, p. 969-983
28. Wu JY, Leung WY, Chang S, Lee B, Zee B, Tong PC, Chan JC: Effectiveness of telephone counselling by a pharmacist in reducing mortality in patients receiving polypharmacy: randomised controlled trial. *Bmj* 333:522, 2006
29. Chan JCN, So WY, Yeung CY, Ko GTC, Lau IT, Tsang MW, Lau KP, Siu SC, Li JKY, Yeung VTF, Leung WYS, Tong PCT: The SURE Study: Effects of Structured versus Usual care on Renal Endpoint in Type 2 diabetes: A randomized multi-centre translational study *Diabetes Care* 32:977-982, 2009
30. Leung WYS, So WY, Tong PCY, Chan NN, Chan JCN: Effects of structured care by a pharmacist-diabetes specialist team in patients with type 2 diabetic nephropathy. *American Journal of Medicine* 118:1414.e1421-1414.e1427, 2005
31. Discher C, Klein D, Pierce L, Levine A, Levine T: Heart failure disease management : Impact on hospital care, length of stay and reimbursement. *Congestive Heart Failure* 9:77-83, 2003
32. Piatt G, Orchard T, Emerson S, Simmons D, Songer T, Brooks M, Korytkowski M, Siminerio L, Ahmad U, Zgibor J: Translating the chronic care model into the community: results from a randomized controlled trial of a multifaceted diabetes care intervention. *Diabetes Care* 29:811-817., 2006
33. Katon WJ, Lin EH, Von Korff M, Ciechanowski P, Ludman EJ, Young B, Peterson D,

Rutter CM, McGregor M, McCulloch D: Collaborative care for patients with depression and chronic illnesses. *N Engl J Med* 363:2611-2620, 2011

34. Chan J, So W, Ko G, Tong P, Yang X, Ma R, Kong A, Wong R, Le Coguiec F, Tamesis B, Wolthers T, Lyubomirsky G, Chow P: The Joint Asia Diabetes Evaluation (JADE) Program: a web-based program to translate evidence to clinical practice in Type 2 diabetes. *Diabet Med* 26:693-699, 2009
35. Ko GT, So WY, Tong PC, Le Coguiec F, Kerr D, Lyubomirsky G, Tamesis B, Wolthers T, Nan J, Chan J: From design to implementation--the Joint Asia Diabetes Evaluation (JADE) program: a descriptive report of an electronic web-based diabetes management program. *BMC Med Inform Decis Mak* 10:26, 2010
36. So WY, Raboca J, Sobrepena L, Yoon KH, Deerochanawong C, Ho LT, Himathongkam T, Tong P, Lyubomirsky G, Ko G, Nan H, Chan J: Comprehensive risk assessments of diabetic patients from seven Asian countries: The Joint Asia Diabetes Evaluation (JADE) program*. *J Diabetes* 3:109-118, 2011
37. Chan JC, So WY, Ma RCW, Tong P, Wong R, Yang XL: The complexity of both vascular and non-vascular complications of diabetes: The Hong Kong Diabetes Registry. *Curr Cardiovasc Risk Rep* 5:230-239, 2011
38. Laakso M, Pyorala K: Age of onset and type of diabetes. *Diabetes Care* 8:114-117, 1985
39. Morrish NJ, Wang S, Stevens LK, Fuller JH, Keen H: Mortality and causes of death in the WHO Multinational Survey of Vascular Diseases in Diabetes. *Diabetologia* 44:S14-21, 2001
40. Kong AP, Yang X, Ko GT, So WY, Chan WB, Ma RC, Ng VW, Chow CC, Cockram CS, Tong PC, Wong V, Chan JC: Effects of treatment targets on subsequent cardiovascular events in Chinese patients with type 2 diabetes. *Diabetes Care* 30:953-959, 2007
41. Yang X, So WY, Kong AP, Ho CS, Lam CW, Stevens RJ, Lyu RR, Yin DD, Cockram CS, Tong PC, Wong V, Chan JC: Development and validation of stroke risk equation for Hong Kong Chinese patients with type 2 diabetes: the Hong Kong Diabetes Registry. *Diabetes Care* 30:65-70, 2007
42. Yang X, So WY, Kong AP, Ma RC, Ko GT, Ho CS, Lam CW, Cockram CS, Chan JC, Tong PC: Development and validation of a total coronary heart disease risk score in type 2 diabetes mellitus. *Am J Cardiol* 101:596-601, 2008
43. Pogue J, Walter SD, Yusuf S: Evaluating the benefit of event adjudication of cardiovascular outcomes in large simple RCTs. *Clin Trials* 6:239-251, 2009
44. Shah BR, Hux JE, Laupacis A, Zinman B, van Walraven C: Clinical inertia in response to inadequate glycemic control: do specialists differ from primary care physicians? *Diabetes Care* 28:600-606, 2005
