# Supplementary material for: Effects of a Technology-Assisted Integrated Diabetes Care Program on Cardiometabolic Risk Factors Among Patients With Type 2 Diabetes in the Asia-Pacific Region: The JADE Program Randomized Clinical Trial
Source: JAMA Netw Open. 2021 Apr 30;4(4):e217557. doi: 10.1001/jamanetworkopen.2021.7557 (PMC8087959; doi:10.1001/jamanetworkopen.2021.7557)
Supplement: Supplement 2. — eFigure 1. Example of JADE Personalized Report eFigure 2. Effects of Intervention (E1+2+3) vs Control (E1+2) on Cardiometabolic Risk Factors in Phase 1 Study, Stratified by National Income Level (Intention-to-Treat and Per-Protocol Analyses) eTable 1. Changes in Medication Usage and the Proportion of Patients Attaining Treatment Targets and Key Performance Indices in the Intention-to-Treat Population eTable 2. Comparison of Baseline Characteristics Between Patients With at Least 12 Months of Observation Who Did and Did Not Return for Repeat Evaluation in Phase 1 Study eTable 3. Comparison of Baseline Characteristics Between Patients With at Least 12 Months of Observation Who Did and Did Not Return for Repeat Evaluation in Phase 2 Study [file jamanetwopen-e217557-s002.pdf]

Supplementary Online Content

Lim LL, Lau ESH, Fu AWC, et al; Asia-Pacific JADE Study Group. Effects of a technology-assisted integrated diabetes care program on cardiometabolic risk factors among patients with type 2 diabetes in the Asia-Pacific region: the JADE Program randomized clinical trial. *JAMA Netw Open*. 2021;4(4):e217557. doi:10.1001/jamanetworkopen.2021.7557

eFigure 1. Example of JADE Personalized Report

eFigure 2. Effects of Intervention vs Control on Cardiometabolic Risk Factors in Phase 1 Study, Stratified by National Income Level (Intention-to-Treat and Per-Protocol Analyses)

eTable 1. Changes in Medication Usage and the Proportion of Patients Attaining Treatment Targets and Key Performance Indices in the Intention-to-treat Population

eTable 2. Comparison of Baseline Characteristics Between Patients With at Least 12 Months of Observation Who Did and Did Not Return for Repeat Evaluation in Phase 1 Study

eTable 3. Comparison of Baseline Characteristics Between Patients With at Least 12 Months of Observation Who Did and Did Not Return for Repeat Evaluation in Phase 2 Study

This supplementary material has been provided by the authors to give readers additional information about their work.

© 2021 Lim LL et al. *JAMA Network Open*.

eFigure 1. Example of JADE Personalized Report

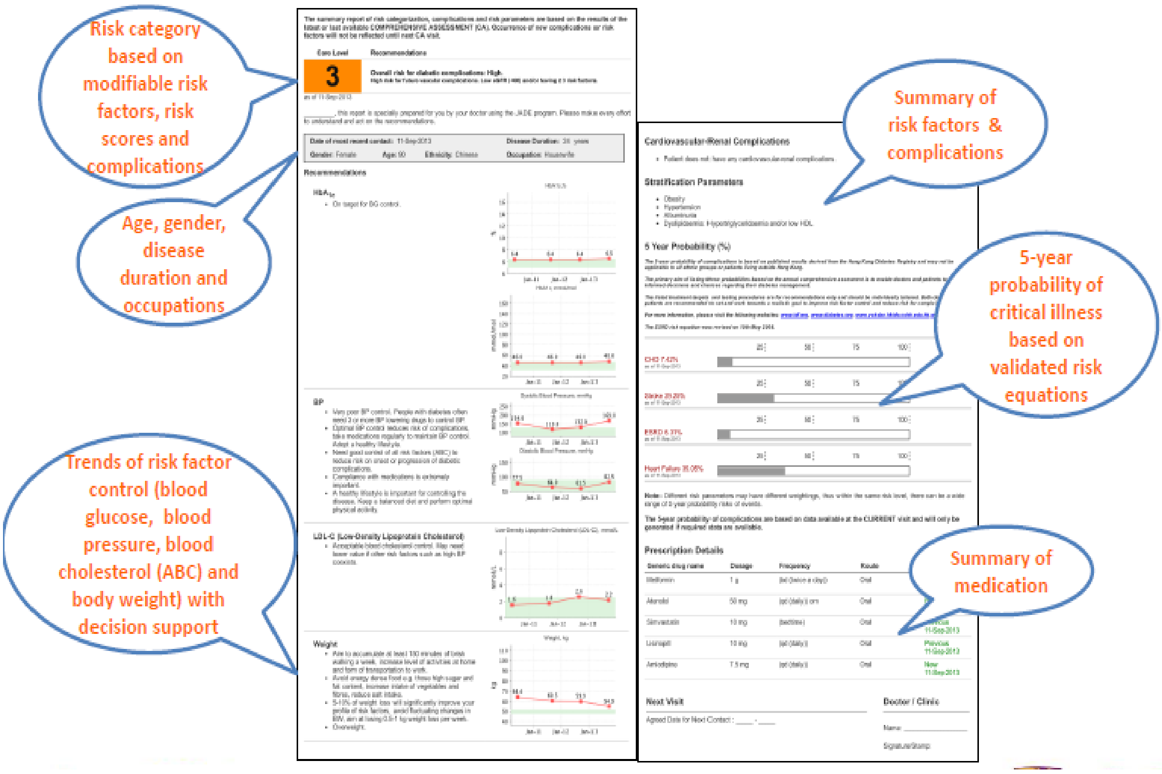

Footnotes: The JADE report, one for the patient and one for the health care professionals, contains following information:

a) Risk level (based on each patient's demographics, medical history, and results of structured evaluation)

b) Trends of key cardiometabolic risk factors

c) 5-year probability of cardiovascular-kidney events (based on validated risk equations)

d) Individualized decision support for both patients and health care professionals. The portal contains 150 short messages triggered by attained levels of key cardiometabolic risk factors, which are frequently conveyed to patients during medical visits. The messages to physicians focus on reminding them to refer patients for education and use of medications to achieve early and optimal control of cardiometabolic risk factors. The messages to patients focus on adherence to treatment and self-management.

© 2021 Lim LL et al. *JAMA Network Open*.

eFigure 2. Effects of Intervention (E1+2+3) vs Control (E1+2) on Cardiometabolic Risk Factors in Phase 1 Study, Stratified by National Income Level (Intention-to-Treat and Per-Protocol Analyses)

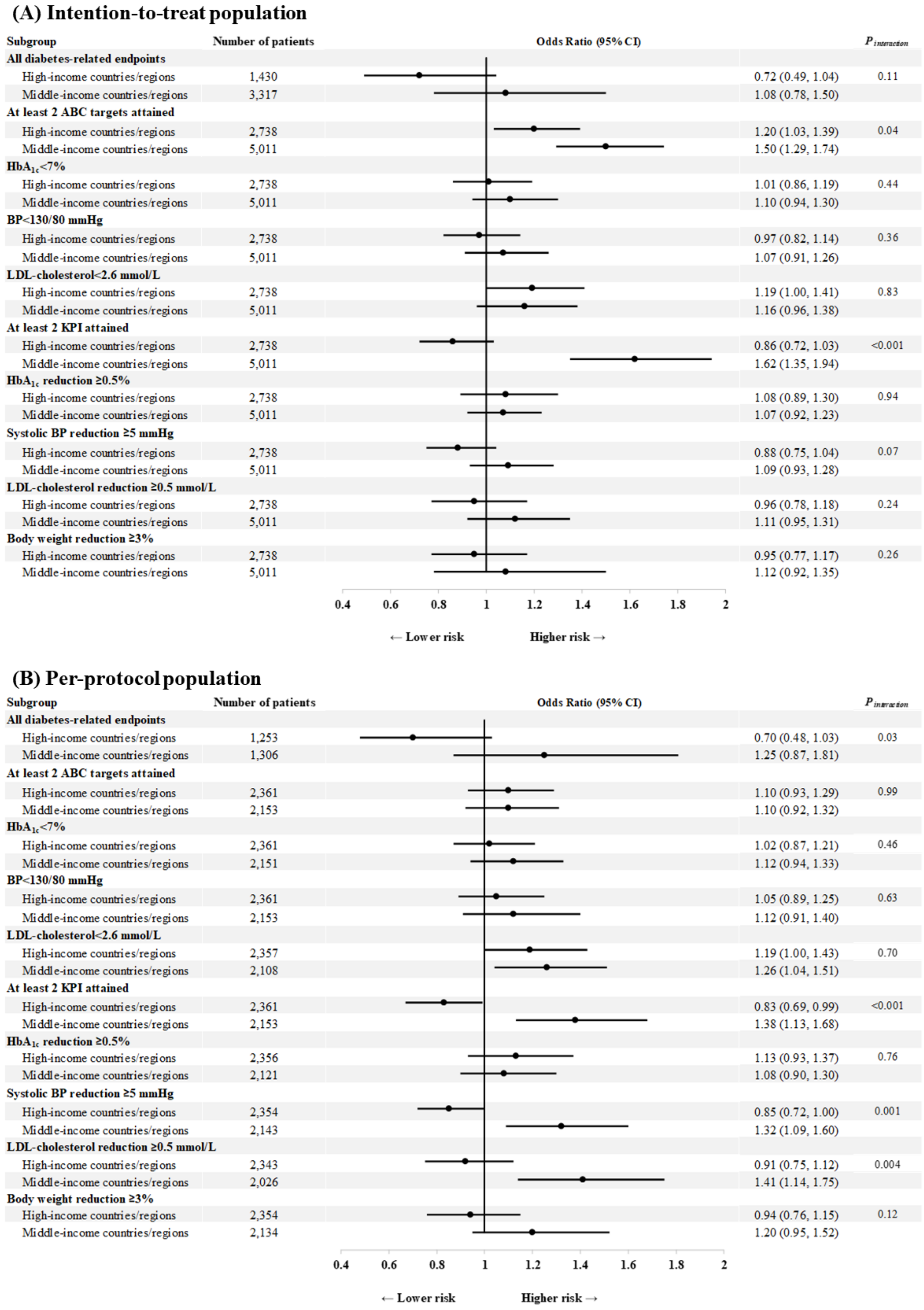

© 2021 Lim LL et al. *JAMA Network Open*.

Footnotes: All logistic regression models were adjusted for country and presented as odds ratio (OR) with 95% confidence interval (CI). The primary outcome was the incidence of a composite of diabetes-associated end points including cardiovascular disease, chronic kidney disease (estimated glomerular filtration rate [eGFR] <60 mL/min/1.73m^2) or end-stage kidney disease (dialysis or eGFR <15 mL/min/1.73m^2), visual impairment/eye surgery, lower-extremity amputation/foot ulcers requiring hospitalization, all-site cancers, and death. The secondary outcomes were 1) attainment of 2 or more primary diabetes-associated targets at 12 months (defined as HbA_{1c} <7%, blood pressure [BP] <130/80 mmHg, and low-density lipoprotein [LDL] cholesterol <2.6 mmol/L [100 mg/dL]) and 2) attainment of 2 or more key performance indexes (KPI) (defined as reduction in HbA_{1c} ≥0.5%, systolic BP ≥5 mmHg, LDL-cholesterol ≥0.5 mmol/L [19 mg/dL], and body weight ≥3%). ABC, Hb**A**$_{1c}$, **B**lood pressure, and LDL-**C**holesterol; E1, structured evaluation guided by the web-based Joint Asia Diabetes Evaluation (JADE) Technology; E2, issue of JADE personalized report for empowerment; E3, ≥2 telephone or face-to-face contacts by nurses for engagement over 12 months. To convert LDL-cholesterol to mg/dL, multiply by 38.67.

© 2021 Lim LL et al. *JAMA Network Open*.

eTable 1. Changes in Medication Usage and the Proportion of Patients Attaining Treatment Targets and Key Performance Indices in the Intention-to-treat Population

	**Phase 1**				**Phase 2**			
	**Baseline**		**At 12 months**		**Baseline**		**At 12 months**	
	**Intervention group**	**Control group**	**Intervention group**	**Control group**	**Intervention group**	**Control group**	**Intervention group**	**Control group**
Total participants, No.	3732	3805	3732	3805	6645	6652	6645	6652
Medications								
Renin-angiotensin system inhibitors	1633 (43.8%)	1659 (43.6%)	1661 (44.5%)	1745 (45.9%)	1641 (24.7%)	1686 (25.3%)	2054 (30.9%)	2045 (30.7%)
Statins	1929 (51.7%)	1887 (49.6%)	2073 (55.5%)	2104 (55.3%)	2004 (30.2%)	1983 (29.8%)	2517 (37.9%)	2461 (37.0%)
Oral glucose-lowering drugs	3118 (83.5%)	3169 (83.3%)	2993 (80.2%)	3168 (83.3%)	5886 (88.6%)	5836 (87.7%)	5430 (81.7%)	5697 (85.6%)
Insulin	919 (24.6%)	1019 (26.8%)	898 (24.1%)	1033 (27.1%)	1869 (28.1%)	1742 (26.2%)	1746 (26.3%)	1676 (25.2%)
Primary targets								
HbA_{1c}<7% (NGSP)	1277 (34.2%)	1284 (33.7%)	1356 (37.0%)	1370 (36.8%)	1325 (19.9%)	1367 (20.6%)	1532 (24.7%)	1557 (25.3%)
Blood pressure <130/80 mmHg	867 (23.2%)	895 (23.5%)	921 (24.9%)	941 (24.9%)	1352 (20.3%)	1381 (20.8%)	1353 (20.8%)	1315 (20.2%)
LDL cholesterol <100 mg/dL	2100 (56.3%)	2090 (54.9%)	2293 (64.0%)	2203 (60.0%)	3184 (47.9%)	3077 (46.3%)	3418 (58.5%)	3179 (54.2%)
At least 2 primary targets attained	1222 (32.7%)	1183 (31.1%)	1314 (35.2%)	1286 (33.8%)	1396 (21.0%)	1400 (21.0%)	1464 (22.0%)	1456 (21.9%)
Key performance indices								
HbA_{1c} reduction ≥0.5%			766 (20.5%)	702 (18.4%)			1017 (15.3%)	802 (12.1%)
Systolic BP reduction ≥5 mm Hg			926 (24.8%)	864 (22.7%)			848 (12.8%)	686 (10.3%)

© 2021 Lim LL et al. *JAMA Network Open*.

	Phase 1				Phase 2			
	Baseline		At 12 months		Baseline		At 12 months	
	Intervention group	Control group	Intervention group	Control group	Intervention group	Control group	Intervention group	Control group
LDL cholesterol reduction ≥19 mg/dL			523 (14.0%)	436 (11.5%)			371 (5.6%)	290 (4.4%)
Body weight reduction ≥3%			409 (11.0%)	364 (9.6%)			471 (7.1%)	284 (4.3%)

Footnotes: HbA_{1c}, glycated hemoglobin; LDL cholesterol, low-density lipoprotein cholesterol; NGSP, National Glycohemoglobin Standardization Program.

© 2021 Lim LL et al. *JAMA Network Open*.

eTable 2. Comparison of Baseline Characteristics Between Patients With at Least 12 Months of Observation Who Did and Did Not Return for Repeat Evaluation in Phase 1 Study

	**Returnees (n=4,514)**	**Non-returnees (n=2,596)**	***P*-value**
Age (years)	61.1±10.8	57.3±11.5	<0.001
Duration of diabetes (years)[a]	10 (5-15)	8 (4-15)	<0.001
Men, %	49.8%	56.0%	<0.001
National income, %			<0.001
High income	52.1%	12.3%	
Low- and middle-income	47.9%	87.7%	
At least college education, %	51.9%	64.5%	<0.001
Current smoker, %	10.2%	7.9%	<0.001
HbA_{1c} (NGSP, %)	7.66±1.56	8.21±1.90	<0.001
Systolic blood pressure (mmHg)	136.0±17.4	130.0±16.1	<0.001
Diastolic blood pressure (mmHg)	79.1±9.9	78.9±8.1	0.25
Total cholesterol (mmol/L)	4.45±0.93	4.60±1.36	<0.001
LDL-cholesterol (mmol/L)	2.54±0.88	2.54±1.11	0.94
HDL-cholesterol (mmol/L)	1.20±0.39	1.09±0.34	<0.001
Triglyceride (mmol/L)[a]	1.43 (1.03-2.01)	1.60 (1.16-2.25)	<0.001
Body mass index (kg/m^2)	26.4±4.6	26.8±4.7	<0.001
Estimated GFR (mL/min/1.73m^2)	79.1±23.3	85.1±22.4	<0.001
CKD, %	20.2%	12.9%	<0.001
CVD, %	17.8%	10.9%	<0.001
Medications			
Renin-angiotensin system inhibitors, %	48.5%	32.0%	<0.001
Statins, %	48.4%	37.0%	<0.001
Oral glucose-lowering drugs, %	84.0%	83.9%	0.001
Insulin, %	24.3%	27.7%	0.001
Primary targets			
HbA_{1c} <7%, %	37.4%	28.7%	<0.001
Blood pressure <130/80 mmHg, %	25.2%	19.9%	<0.001
LDL-cholesterol <2.6 mmol/L (100 mg/dL), %	58.9%	57.5%	0.29
At least 2 primary targets attained, %	34.8%	25.2%	<0.001

Footnotes: The non-returnees referred to those with at least 12 months of observation but did not return for repeat evaluation three months before or after the prespecified date. Data are expressed as mean±standard deviation, number (percentage), or median (interquartile range)[a]. CKD, chronic kidney disease; CVD, cardiovascular disease; GFR, glomerular filtration rate; HbA_{1c}, glycated hemoglobin; HDL-cholesterol, high-density lipoprotein cholesterol; LDL-cholesterol, low-density lipoprotein cholesterol; NA, not applicable; NGSP, National Glycohemoglobin Standardization Program; SMBG, self-monitoring blood glucose. To convert triglyceride to mg/dL, multiply by 88.5. To convert LDL-cholesterol to mg/dL, multiply by 38.67.

© 2021 Lim LL et al. *JAMA Network Open*.

eTable 3. Comparison of Baseline Characteristics Between Patients With at Least 12 Months of Observation Who Did and Did Not Return for Repeat Evaluation in Phase 2 Study

	**Returnees (n=3,901)**	**Non-returnees (n=3,874)**	***P*-value**
Age (years)	54.4±10.8	54.2±11.5	0.31
Duration of diabetes (years)[a]	8 (4-14)	7 (3-13)	<0.001
Men, %	59.7%	55.8%	0.001
At least college education, %	52.6%	56.2%	0.002
Current smoker, %	8.6%	10.1%	0.03
HbA_{1c} (NGSP, %)	8.23±1.99	8.58±2.00	<0.001
Systolic blood pressure (mmHg)	130.0±15.6	130.0±16.4	0.96
Diastolic blood pressure (mmHg)	79.2±8.4	79.0±8.7	0.43
Total cholesterol (mmol/L)	4.53±1.18	4.76±1.35	<0.001
LDL-cholesterol (mmol/L)	2.49±1.12	2.74±1.18	<0.001
HDL-cholesterol (mmol/L)	1.10±0.34	1.13±0.44	0.002
Triglyceride (mmol/L)[a]	1.60 (1.18-2.16)	1.59 (1.18-2.15)	0.94
Body mass index (kg/m^2)	27.5±5.0	27.4±5.1	0.27
Estimated GFR ($mL/min/1.73m^2$)	83.8±21.4	82.6±22.8	0.03
CKD, %	12.0%	12.5%	0.49
CVD, %	15.3%	15.5%	0.82
Medications			
Renin-angiotensin system inhibitors, %	25.1%	27.7%	0.01
Statins, %	29.6%	34.3%	<0.001
Oral glucose-lowering drugs, %	93.6%	84.0%	<0.001
Insulin, %	30.2%	30.1%	1.00
Primary targets			
HbA_{1c} <7%, %	29.9%	21.5%	<0.001
Blood pressure <130/80 mmHg, %	26.3%	21.9%	<0.001
LDL-cholesterol <2.6 mmol/L (100 mg/dL), %	62.8%	49.8%	<0.001
At least 2 primary targets attained, %	31.2%	17.7%	<0.001

Footnotes: The non-returnees referred to those with at least 12 months of observation but did not return for repeat evaluation three months before or after the prespecified date. Data are expressed as mean±standard deviation, number (percentage), or median (interquartile range)[a]. CKD, chronic kidney disease; CVD, cardiovascular disease; GFR, glomerular filtration rate; HbA_{1c}, glycated hemoglobin; HDL-cholesterol, high-density lipoprotein cholesterol; LDL-cholesterol, low-density lipoprotein cholesterol; NA, not applicable; NGSP, National Glycohemoglobin Standardization Program; SMBG, self-monitoring blood glucose. To convert triglyceride to mg/dL, multiply by 88.5. To convert LDL-cholesterol to mg/dL, multiply by 38.67.

© 2021 Lim LL et al. *JAMA Network Open*.
