# Supplementary material for: Effects of a Technology-Assisted Integrated Diabetes Care Program on Cardiometabolic Risk Factors Among Patients With Type 2 Diabetes in the Asia-Pacific Region: The JADE Program Randomized Clinical Trial
Source: JAMA Netw Open. 2021 Apr 30;4(4):e217557. doi: 10.1001/jamanetworkopen.2021.7557 (PMC8087959; doi:10.1001/jamanetworkopen.2021.7557)
Supplement: Supplement 3. — Data Sharing Statement [file jamanetwopen-e217557-s003.pdf]

Data Sharing Statement

Lim LL, Lau ESH, Fu AWC, et al; Asia-Pacific JADE Study Group. Effects of a Technology-Assisted Integrated Diabetes Care Program on Cardiometabolic Risk Factors Among Patients With Type 2 Diabetes in the Asia-Pacific Region: the JADE Program randomized clinical trial. *JAMA Netw Open*. Published April 30, 2021. doi:10.1001/jamanetworkopen.2021.7557

Data
Data available: No

Additional Information
Explanation for why data not available: Data cannot be shared publicly as we did not have patients' consent to release the data in the public domain for open, unrestricted access. Researchers who are interested and meet the criteria for research access to our data may apply via Asia Diabetes Foundation (enquiry@adf.org.hk).
